# Mitochondrial haplotype and sex modulate responses to endurance exercise training

**DOI:** 10.1113/JP288330

**Published:** 2025-05-31

**Authors:** Bumsoo Ahn, Tianhao Wei, Ryan Pettit-Mee, Eunyoung Kim, Robert V. Musci, Jonathan Wanagat, Hoang Van M. Nguyen, Arlan Richardson, Hyunyoung Kim

**Affiliations:** 1 Department of Internal Medicine, Section on Gerontology and Geriatric Medicine, Wake Forest School of Medicine, Winston-Salem, North Carolina, USA; 2 Sticht Center for Healthy Aging and Alzheimer’s Prevention, Wake Forest School of Medicine, Winston-Salem, North Carolina, USA; 3 Department of Health and Human Sciences, Frank R Seaver College of Science and Engineering, Loyola Marymount University, Los Angeles, California, USA; 4 Divisions of Geriatrics, Department of Medicine, University of California Los Angeles, Los Angeles, California, USA; 5 Veterans Administration Greater Los Angeles Healthcare System, Los Angeles, California, USA; 6 Department of Nutritional Sciences, University of Oklahoma Health Sciences, Oklahoma City, Oklahoma, USA; 7 Oklahoma City VA Medical Center, Oklahoma City, Oklahoma, USA; 8 Department of Biochemistry & Physiology, University of Oklahoma Health Sciences, Oklahoma City, Oklahoma, USA

**Keywords:** endurance exercise, mitochondrial bioenergetics, mitochondrial DNA, mitochondrial haplotype, motor co-ordination, neuromuscular junction, sex

## Abstract

Heterogeneity in the response to exercise training is widely demonstrated in the literature. Although the variability in exercise acclimation is not entirely understood, a large portion of exercise response variability is attributable to genetic heritability potentially due to inherited maternal mitochondrial characteristics. Humans exhibit a heterogenous genome and mitochondrial haplotype; however much of the preclinical research proposed to investigate molecular transducers of exercise has been implemented using mouse models that lack mitochondrial and nuclear genomic diversity. Leveraging a novel rat model of heterogeneous genome, OKC-HET rats, we investigated the impact of mitochondrial (mt) haplotype on exercise training. We hypothesized that rats with divergent mitochondrial genomes will respond differently to endurance exercise training. OKC-HET rats aged 18–19 months old were subjected to 8 weeks of voluntary wheel running as their endurance exercise training programme. We found mt haplotype-specific effects on responses to endurance exercise and motor co-ordination, which were consistent with mitochondrial bioenergetics and markers of oxidative stress. Mitochondrial copy number and the expression of mitochondrial proteins were similar between the two mt haplotypes, suggesting intrinsic alterations of mitochondrial functions by the two distinct mitochondrial genomes. Motor co-ordination and fragmentation of acetylcholine receptors were also affected by mitochondrial haplotype. The mt haplotype effects on training responses were specific to biological sex also. Collectively we report that mitochondrial haplotype significantly affects responses to endurance exercise in a sex-specific manner.

## Graphical Abstract

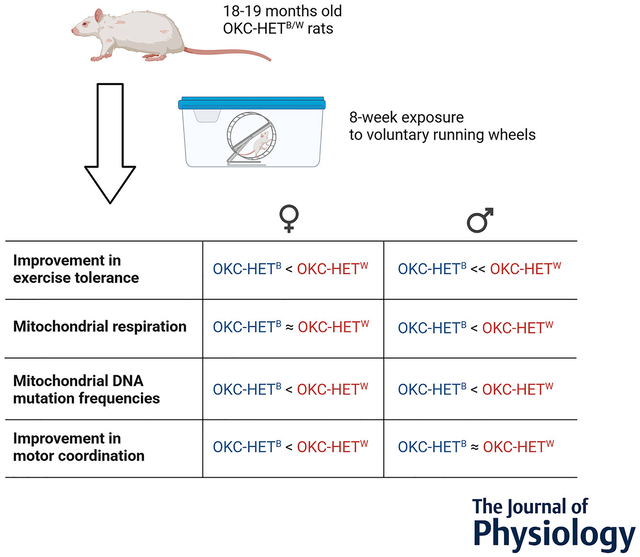

Using OKC-HET^B/W^ rats we demonstrate that mitochondrial haplotype influences training responses of endurance exercise. Overall OKC-HET^W^ rats showed greater responses than OKC-HET^B^ in exercise tolerance, mitochondrial bioenergetics, mitochondrial DNA (mtDNA) mutation frequencies and motor co-ordination. Biological sex also modulated training responses.

## Introduction

Ageing is the greatest risk factor for most chronic diseases and geriatric syndromes. The traditional view is that ageing effects are not modifiable, but this notion has been challenged by the geroscience hypothesis (Kritchevsky & Justice, 2020), proposing that biological ageing can be targeted through interventions. An unmet need in the field of ageing biology is the development of therapeutic strategies that effectively mitigate the negative consequences of ageing. Exercise remains one of the most promising interventions to combat the biological ageing, to delay physiological decline and mobility disability (Garatachea et al., 2015). A critical opportunity lies in identifying the factors that contribute to the variability in exercise responsiveness, which could lead to improved health outcomes in older adults.

Interindividual differences in responses to the same exercise regimen have been recognized in the scientific literature for nearly 40 years (Despres et al., 1988; Prudhomme et al., 1984; Simoneau et al., 1986). For example the Health, Risk Factors, Exercise Training and Genetics (HERITAGE) Family Study explored genetic influence on changes in cardiorespiratory fitness (VO_2max_) after a standardized exercise training programme. Recent estimates indicate that interindividual variation in VO_2max_ ranges from −4.7% to 47.8%, with genetic heritability accounting for 47% of this variation (Sarzynski et al., 2022). Additional factors contributing to these differences in VO_2max_ response include age and sex. A well-established approach for enhancing genetic diversity is the four-way cross-strategy, first applied by Roderick to produce a genetically heterogeneous mouse population (Roderick, 1963). Miller and colleagues later used a similar method to create UM-HET3 mice (Miller et al., 1999), which are now widely used in ageing research, including in the National Institute of Aging’s Intervention Testing Program. The primary advantage of a four-way cross is its reproducibility; researchers can generate similar populations by sourcing the parental strains from commercial vendors. Although UM-HET3 mice exhibit nuclear genetic heterogeneity, the breeding scheme results in all offspring inheriting the same BALB/c mitochondrial genome (Miller et al., 1999).

Genetic heritability in exercise responses is well documented (Sarzynski et al., 2022), but very little attention has been paid to the role of genetic variability in the mitochondrial haplotype. This is surprising and represents a barrier in the field because mitochondria are crucial for response to endurance training and are responsible for transforming most of the cellular energy needed to meet metabolic demands. Exercise training induces changes to the mitochondria (Oliveira & Hood, 2019), allowing for greater energy transformation during physical activity and improved endurance capacity. Others reported increased mitochondrial dynamics, mitophagy (Drake et al., 2019) and calcium retention capacity (Yoo et al., 2019), which are critical during exercise. Furthermore disrupting mitochondrial function seems to blunt the response to exercise training. Combining the anti-diabetic drug metformin with endurance exercise training blunted the improvements in the maximum oxygen consumption (VO_2_max), insulin sensitivity and aerobic capacity, elicited by endurance exercise (Konopka et al., 2019; Malin et al., 2012). This is likely due to metformin’s inhibitory effect on mitochondria complex I activity, thereby modulating the response to exercise. Similar results were reported when exercise training was combined with statins, which also inhibit mitochondrial bioenergetics (Mikus et al., 2013). More recently the Molecular Transducers of Physical Activity Consortium (MoTrPAC) highlighted that mitochondrial biogenesis and oxidative phosphorylation are key molecular pathways highly affected by endurance exercise (MoTr et al., 2024).

The current laboratory mouse models used in biomedical research lack mitochondrial diversity, which is a result of the traditional inbred strains of laboratory mice originating from a single female Mus musculus domesticus mouse (Bayona-Bafaluy et al., 2003; Ferris et al., 1982; Yu et al., 2009). A study sequencing mitochondrial DNA in 52 common inbred mouse strains revealed that 18 strains shared identical mitochondrial genomes, with the others differing by only 1–4 nucleotide variations (Yu et al., 2009). The importance of divergent mitochondrial genomes on a variety of physiological outcomes was shown by the Ballinger group who generated mitochondrial nuclear exchange mouse models (MNX) in which the mitochondrial DNA (mtDNA) from the C3H mouse was merged with the C57BL/6 nuclear background and vice versa (Fetterman et al., 2013). They showed that the mitochondrial genetic background modulated a variety of functions and pathways in various pathological conditions and diseases (Betancourt et al., 2014; Dunham-Snary et al., 2018; Fetterman et al., 2013; Sammy et al., 2021). However the mitochondrial genomes from C57BL/6 and C3H mice differ by only five nucleotides. This lack of mitochondrial diversity reduces the translatability of these models to humans, who possess much more diverse mitochondrial genomes (Ingman et al., 2000; Merriwether et al., 1991; Yu et al., 2009). Poor translatability is also evident in common chronic diseases such as cancer (Mak et al., 2014) and Alzheimer’s disease (Carter et al., 2020; Shineman et al., 2011), where pharmacological interventions tested in mouse models rarely succeed in humans. In contrast rats offer a more suitable model for ageing and biomedical research, as they have more diverse mitochondrial genomes (Sathiaseelan et al., 2023) and exhibit age-related pathologies and sex-based survival advantages similar to humans (Pereira et al., 2009; Snyder et al., 2016). Maximal OCR (i.e. VO_2max_) in humans ranges from 30 to 60 ml/kg/min (Bassett & Howley, 2000), which is significantly closer to that of rats, ranging from 60 to 80 ml/kg/min (Bedford et al., 1979; Rodrigues et al., 2007), than of mice, which have a VO_2max_ of 140–180 ml/kg/min (Fernando et al., 1993; Schefer & Talan, 1996). Furthermore fibre-type composition and size of human myofibres are closer to rats than mice (Delp & Duan, 1996; Manttari & Jarvilehto, 2005; Staron et al., 2000). To enhance mitochondrial haplotype diversity in a model with physiological characteristics similar to humans during exercise, we used the recently developed OKC-HET^B/W^ rats, which have divergent mitochondrial genomes inherited from Brown Norway and Wistar Kyoto (WKY) rats. The mtDNA sequences of OKC-HET^B^ and OKC-HET^W^ rats differ by 94 nucleotides (Sathiaseelan et al., 2023), making them ideal for investigating mitochondrial genome heterogeneity in response to endurance exercise.

In this study we tested whether distinct mitochondrial haplotypes influence the responses of endurance exercise training. OKC-HET rats, aged 18–19 months, were used to test the effects of endurance exercise before the onset of sarcopenia. Our data demonstrate that the OKC-HET^B/W^ rats responded to endurance exercise in a mitochondrial haplotype- and sex-specific manner, highlighting the significant effects of the mitochondrial haplotype and biological sex on exercise training responses.

## Materials and methods

### Ethical approval

Animal procedures were performed according to all national and local guidelines and regulations, the Guide for the Care and Use of Laboratory Animals, the ARRIVE guidelines (Percie du Sert et al., 2020). Breeding and animal care were in accordance with the National Institutes of Health’s guidelines and approved by the Institutional Animal Care and Use Committee at the Oklahoma City VA Medical Centre. All experiments and procedures of this study were approved by the Institutional Animal Care and Use Committee of the Atrium Health Wake Forest Baptist Medical Centre (A22-027).

### Study design

OKC-HET^B/W^ rats, aged 18–19 months, were used to investigate the effects of mitochondrial haplotype in response to endurance exercise. We used a total of 71 rats (35 males and 36 females). Among the males 20 were of the OKC-HET^B^ strain and 15 were of the OKC-HET^W^ strain. Among the females 22 were of the OKC-HET^B^ strain and 14 were of the OKC-HET^W^ strain. Rats were single-housed in cages with voluntary running wheels for 8 weeks. Exercise tolerance and motor co-ordination were measured before and after the voluntary wheel running (VWR) activities. End-point measures include muscle mass, contractile properties, mitochondrial OCR and reactive oxygen species (ROS) generations. We performed immunofluorescence analyses to image Ach receptors (AchR) and immunoblots to measure mitochondrial protein expressions. We also measured mtDNA copy number and deletion mutation frequency using lower-limb muscles.

### Animals

OKC-HET^B/W^ rats were generated through a four-way cross between Brown Norway, Fischer344, Lewis and Wistar Kyoto rat strains at the Oklahoma City VA Medical Centre. The resulting OKC-HET^B/W^ rats have identical nuclear genotype yet diverse mitochondrial genotypes; the OKC-HET^B^ strain has the mitochondrial genotype of the Brown Norway strain, and the OKC-HET^W^ strain has the mitochondrial genotype of the Wistar Kyoto strain. Animals were group-housed with the number per cage dependent on the size of the rats. At 10–13 months of age rats were transferred from the Oklahoma City VA Medical Centre to the Wake Forest University School of Medicine barrier facility under the supervision of the Animal Research Program. Male and female OKC-HET^B/W^ rats aged 18–19 months were single-housed in a cage with wheel. All rats were caged in pathogen-free conditions and provided with free access to standard chow and water and maintained on a 12 h light/dark cycle. Animals were maintained in a temperature-controlled room (21°C ± 1°C).

### Voluntary wheel running

All rats were housed in standard cages equipped with special cage tops and voluntary running wheels (Ugo Basile, Gemonio, Italy). Rats were individually housed to facilitate free access to the wheel. The rotation of the running wheel was detected by a magnet and measured via a small counter located on the wheel. Over the 8 weeks wheel rotation number was recorded every morning, and body mass of each rat was measured every 2 weeks. Exercise tolerance test and motor co-ordination (treadmill and rotarod tests) were completed the week before and after the training period. In this analysis n represents data from individual rat.

### Graded exercise test

Exercise tolerance was determined using a modified version of a previously established treadmill test protocol (Ahn et al., 2019). Treadmill running tests were performed on single-lane treadmill set (Panlab LE8700, Harvard Bioscience, Holliston, MA, USA) at a 5% grade incline. Before being tested rats were familiarized with the treadmill for four consecutive days; on the first day rats were placed on a non-moving treadmill for 10 min. Over the next 3 days rats were placed on the treadmill with a starting belt speed of 8 cm/s. After each minute the belt speed was increased by 1 cm/s until the rats ran at 12 cm/s for 1 min, when familiarization was terminated. On test day rats were placed on the treadmill with a starting belt speed of 12 cm/s for 5 min, then the speed would gradually increase by 5–7 cm/s every 5 min. Exhaustion was defined as failure to run on the treadmill despite gentle physical prodding from behind. The treadmill running end time and distance were recorded. In this analysis n represents data from individual rat.

### Motor co-ordination

Motor co-ordination was determined with the accelerating rotarod protocol using a Rota Rod equipment (Rotamex 4/8, Columbus Instruments, OH, USA). Before being tested rats were familiarized with the rotarod apparatus for 1 day, during which the rats were placed on and engaged the rod with 5 rpm for 60 s, which was repeated thrice, with a 2-min rest between trials. On test days rats were placed on and engaged the rod with a starting speed of 5 rpm that gradually increased to 35 rpm over 7 min by linear acceleration. The same test was repeated for three trials, with a 2-min rest between trials. Rat latency to fall was measured for three trials per day over 3 consecutive days after the familiarization day. The average time until falling each day was used for analysis. In this analysis n represents data from individual rat.

### Animal killing

Two days after completion of the last exercise performance and motor co-ordination tests, rats were killed by carbon dioxide asphyxiation at a fill rate of 40%–50% displacement of the chamber per minute. After death was confirmed via cervical dislocation and toe pinching using forceps, laparotomy was performed for vital tissue dissections. We isolated lower-limb muscle tissues and performed functional assays using fresh tissues below. The rest of the tissues were snap frozen immediately in liquid nitrogen and stored in −80° freezer until further analyses.

### Acetylcholine receptor staining and analysis

AchR morphologies were visualized and determined using a modified version of the previously established staining and analysis protocols (Ahn et al., 2022; Kim et al., 2024). Gastrocnemius muscle was obtained during dissection, and small pieces of muscle tissues were collected. In a Petri dish filled with cold phosphate-buffered saline (PBS) connective tissues were removed from the muscle samples. Muscle samples were first fixed with 4% formaldehyde for 30 min at room temperature with vigorous shaking. After fixation muscle samples were washed thrice for 5 min in PBS at room temperature. The muscle samples were then stained with a bungarotoxin antibody (Alpha-Bungarotoxin, #B13422, Invitrogen, Carlsbad, CA, USA) for 30 min at room temperature. After staining the muscle samples were transferred onto slides, mounted with mounting medium and covered with cover glass, with 2–3 sample tissues placed on each slide. AchR images were captured using a confocal microscope (Olympus Evident FV4000, Olympus, Tokyo, Japan). Magnification at 10× and Z-stacks were applied to capture the complete 3D structure of the AchRs. For data analysis n represents quantified results of individual AchRs from 5–12 images in 4–5 rats/group. Five to fifteen AchRs were analysed from each image. The area of the AchRs was measured for the forward-facing AchRs on each image using Image J software. The fragmentation of the AchRs was categorized by manually counting the pieces of each forward-facing AchRs.

### Fibre permeabilization

Preparation for skeletal muscle fibre permeabilization was performed as previously described (Ahn et al., 2018; Laitano et al., 2016). Briefly a small piece (~3–5 mg) of red gastrocnemius muscle was carefully dissected, and we separated fibres in ice-cold BIOPS media, containing 10 mM Ca-EGTA buffer, 0.1 μM free calcium, 20 mM imidazole, 20 mM taurine, 50 mM K-MES, 0.5 mM DTT, 6.56 mM MgCl_2_, 5.77 mM ATP, 15 mM phosphocreatine, pH 7.1. The muscle bundle was permeabilized in saponin solution (30 μg/ml) for 30 min followed by 5 min washes (3×) in ice-cold MiR05 containing (in mM) 0.5 EGTA, 3 MgCl_2_•6H_2_O, 60 potassium-lactobionate, 20 taurine, 10 KH_2_PO_4_, 20 HEPES, 110 Sucrose and 1g/l BSA, pH 7.1.

### Assessment of mitochondrial respiration

OCR and the rate of mitochondrial hydrogen peroxide production were simultaneously determined using high-resolution respirometer (O2k, OROBOROS Instruments, Innsbruck, Austria) and a previously described method (Ahn et al., 2019). OCR was determined using an oxygen sensor. Measurements were performed on permeabilized fibres in MiR05 media at 37°C, a temperature historically used in permeabilized myofibre experiments (Gnaiger, 2009) that allow comparisons with results from other studies (Kuznetsov et al., 2008; Picard et al., 2010). Rates of respiration were measured using sequential additions of substrates and inhibitors as follows: octanoylcarnitine (0.5 mM), malate (0.1 mM), ADP (2.5 mM), glutamate (10 mM), malate (2 mM), succinate (10 mM), rotenone (0.5 μM), antimycin A (2.5 μM). Our experiments are designed to stimulate specific pathways that drive mitochondrial oxygen consumption, including fatty acid oxidation-, NADH- and succinate-linked pathways, as described previously (Doerrier et al., 2018). All respiratory rates were corrected for residual oxygen consumption measured after inhibition of the mitochondrial electron transport system by antimycin A. Data were normalized to milligrams of muscle bundle wet mass. In this analysis n represents data from individual rat.

### *In vitro* contractile force assessment

Force generation of extensor digitorum longus (EDL) was assessed *in vitro* as previously described (Roberts et al., 2013). Female rats were killed using gaseous CO_2_, and one EDL muscle was immediately excised and prepared for functional assays in a bicarbonate-buffered solution gassed with a mixture of 95% O_2_ and 5% CO_2_ at room temperature. We placed the EDL muscle in an organ bath containing bicarbonate-buffered solution at room temperature and determined the length that induces maximal twitch force, i.e. optimal length (*L*_O_). Muscles were allowed 10 min of thermal equilibration at 32°C, a temperature close to human lower-limb muscle (Chiesa et al., 2016). Measurements of force-frequency were then initiated. In all electrical stimulations a supramaximal current (600–800 mA) of 0.25 ms pulse duration was delivered through a stimulator (701C, Aurora Scientific, Aurora, Canada). Five minutes after the force-frequency protocol the EDL muscle was stimulated to fatigue during isometric contractions (pulse frequency 50 Hz, train duration 500 ms, train rate 0.25 Hz). All data were recorded and analysed using commercial software (DMC and DMA, Aurora Scientific). Specific force (N/cm^2^) was calculated by multiplying the ratio of fibre length to muscle length. In this analysis n represents data from individual rat.

### MtDNA copy number and deletion frequency analyses

MtDNA copy number and deletion mutation frequency were measured using previously validated digital PCR approaches (Herbst et al.). Total DNA was extracted from muscle powder using a DNA extraction kit (GenFind V3, Beckman Coulter, Lifesciences, Raleigh-Durham, CA, USA) and eluted in 10 mM Tris-EDTA buffer, pH 8. Total DNA quality and quantity was assessed using spectrophotometry at A230, A260 and A280 (Nanodrop 2000 Spectrophotometer, ThermoScientific, Waltham, MA, USA), fluorometry (Qubit 2.0 Fluorometer, ThermoScientific, Waltham, MA, USA) and integrity examined by gel electrophoresis or tapestation (TapeStation 4200, Agilent, Santa Clara, CA, USA). A 5-prime nuclease cleavage assay and droplet-based digital PCR (ddPCR) were used to quantify copy numbers for nuclear DNA, total mtDNA and mtDNA deletion mutations with specific primer/probe sets for each, as previously described (Herbst et al.). Samples were diluted to the manufacturer’s recommended target range (20–2000 target copies per microlitre). Digital PCR cycling conditions were polymerase activation at 95°C for 10 min, 40 cycles of denaturation at 94°C for 30 s and annealing/extension at 60°C for 2 min. Reaction threshold and target copy number per microlitre were determined using QuantStudio 3D Analysis Suite Cloud Software (ThermoFisher, Waltham, MA, USA). The same cycling conditions were used for direct quantitation of the major arc deletions by ddPCR but with 60 cycles. Researchers performing the mtDNA assays were blinded to sample characteristics. In this analysis n represents data from individual rat.

### Western blot

Western blots were performed using SDS-PAGE electrophoresis system. Gastrocnemius tissue lysates were prepared in a Ripa buffer containing 50 mM Tris-Cl (pH 7.4), 1 mM EDTA, 0.5 mM EGTA, 1% Triton X-100, 0.1% sodium deoxycholate, 0.1% SDS, 140 mM NaCl. Equal amounts (10–20 μg) of protein samples were loaded on a gel (4%–20% Mini-Protean TGX Precast protein gel, Bio-Rad, Hercules, CA, USA) with 1× tris/glycine/SDS buffer. Proteins were transferred onto PVDF membranes using Trans-Blot Turbo Mini PVDF Transfer Packs (0.2 μm pore size). Blots were imaged using Odyssey DLx imaging system and quantified by Image J and Empiria Studio softwares (LI-COR, Lincoln, NE, USA). Signal intensities were used to normalize total protein loading. Total OXPHOS Rodent Antibody Cocktail (ab110413, Abcam, Cambridge, UK) was used to measure expressions of proteins of individual mitochondrial complexes, and 4-hydroxynonenol (4-HNE, ab46545, Abcam, Cambridge, UK) antibody was used to determine levels of lipid peroxidation. In this analysis n represents data from individual rat.

### Statistical analyses

Prism 10 (GraphPad Software, San Diego, CA, USA) was used for graphing and statistical analyses. After normality was confirmed, paired and unpaired *t* tests were performed to compare means between groups. Multiple unpaired *t* tests were used to compare means of VWR distance and mitochondrial respiration. Holm-Sidak method was used to account for the false discovery rate. Two-way ANOVA (genotype × training) was used to compare means of treadmill distance between groups followed by *post hoc* tests. Values are presented as mean ± SD.

## Results

### Voluntary running activities are sex dependent but mitochondrial haplotype independent

**Figure 1.**
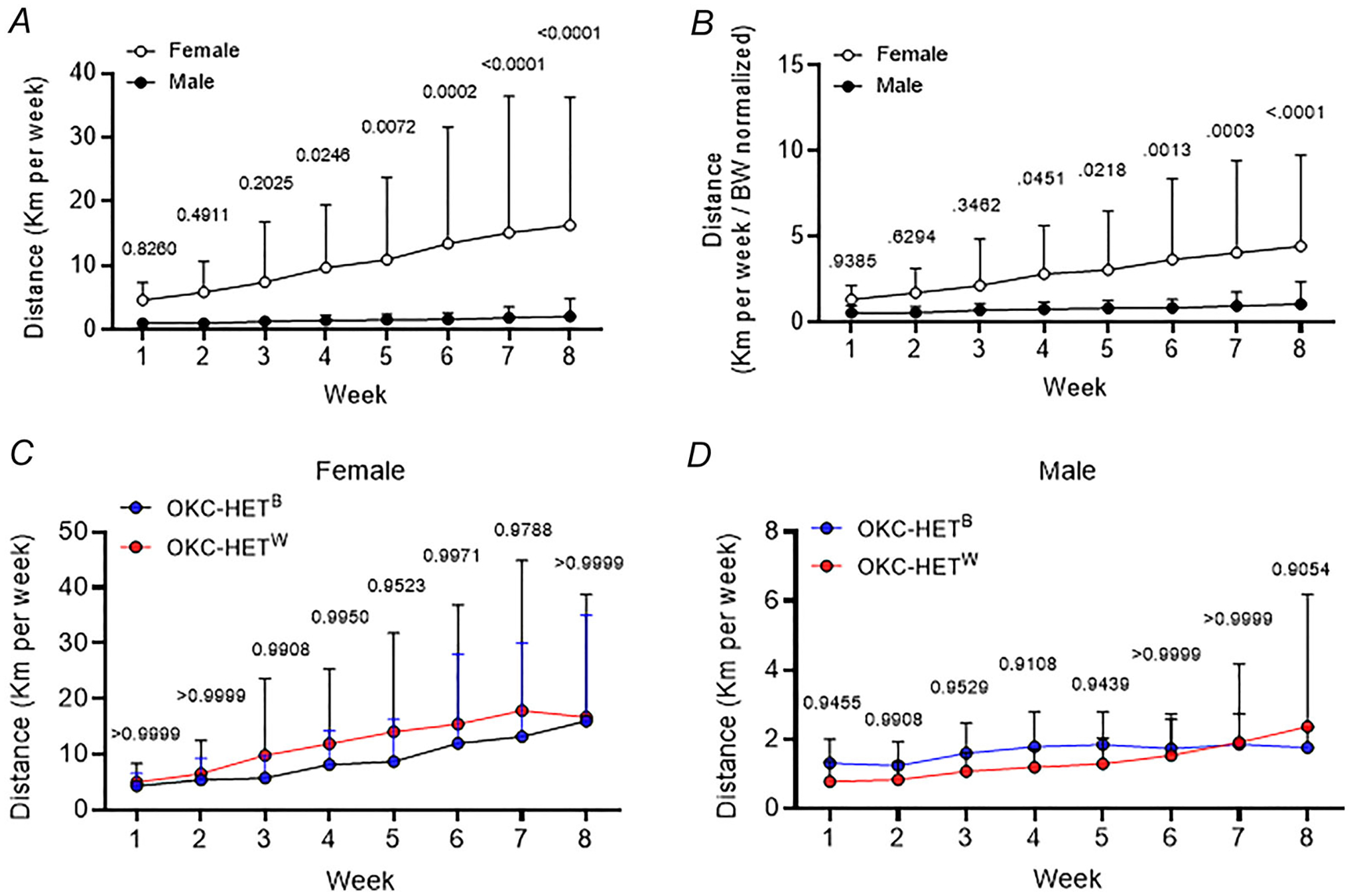
The daily activities of voluntary wheel running of OKC-HETB and OKC-HETW rats *A*, the voluntary running distance (kilometres per day) of male and female OKC-HET rats. *n* = 27–29. *B*, the voluntary running distance (kilometres per day normalized by body mass) in male and female OKC-HET rats. *n* = 27–29. *C*, *D*, voluntary wheel running (VWR) distance of female and male OKC-HETB and OKC-HETW rats. *n* = 12–14. *n* indicates data from each rat. Multiple unpaired *t* tests were used to compare means. Holm-Sidak method was used to account for the false discovery rate. Values are presented as mean ± SD.

**Figure 2.**
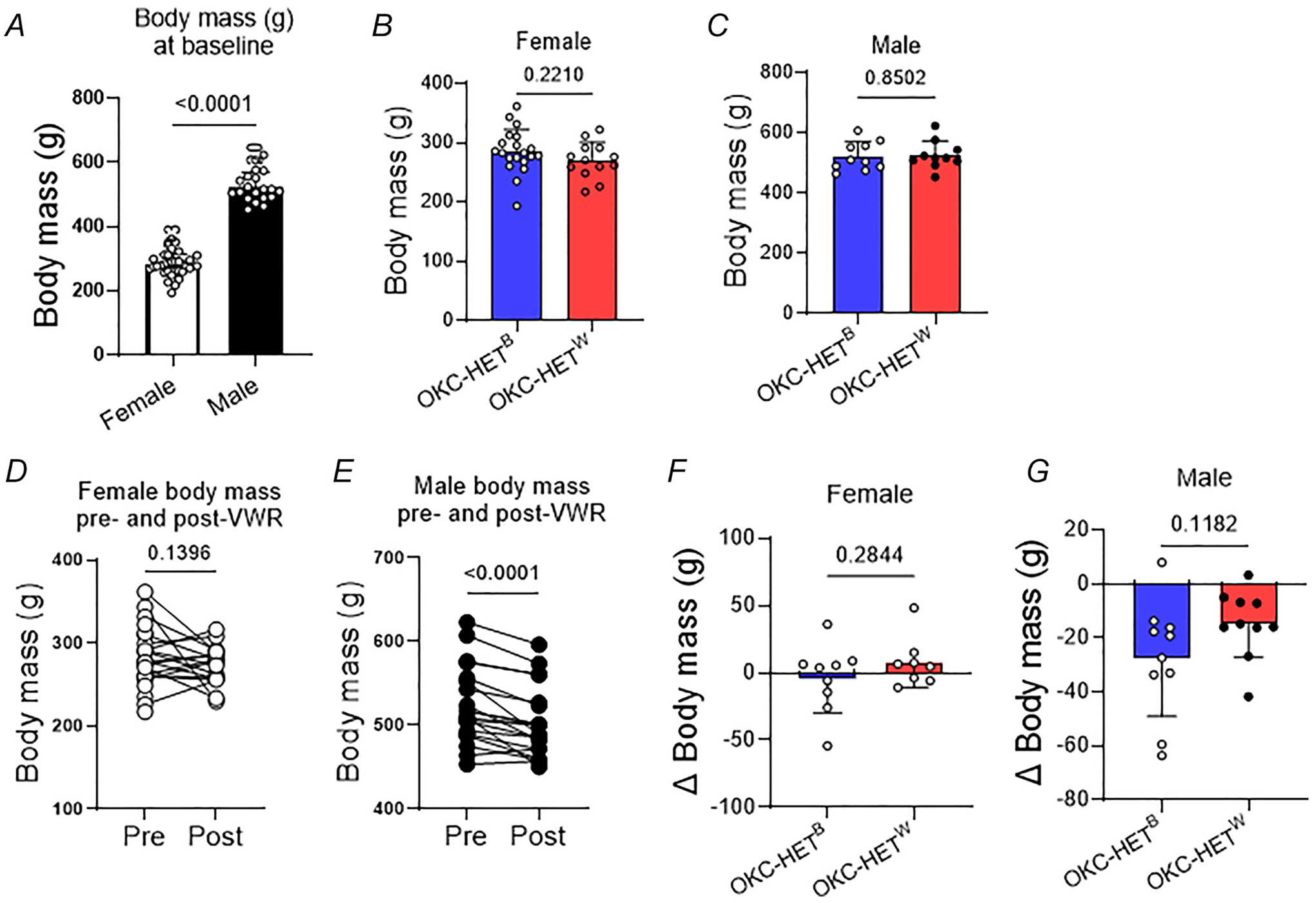
Baseline body mass and body mass changes after 8 weeks of voluntary running activity *A*, baseline body mass of male and female OKC-HET rats. *n* = 20–33. Baseline body mass of female (*B*) and male rats (*C*). *n* = 10–20. Changes in the body mass of female (*D*) and male (*E*) OKC-HET rats after 8 weeks of voluntary wheel running (VWR) activities. *n* = 17–24. Post-training body mass changes of OKC-HET^B^ and OKC-HET^W^ rats for female (*F*) and male (*G*). *n* indicates data from each rat. Values are presented as mean ± SD. Paired and unpaired *t* tests.

We investigated the responses to VWR activities in OKC-HET rats. Over the 8-week training period weekly running distance at baseline was substantially higher in females than in the males. By the end of the 8-week training running distance had increased approximately 2-fold in males but 3.5-fold in females (Fig. 1A, *n* = 27–29 rats). Given the substantial differences in male and female rats’ body mass (Fig. 2A, *n* = 20–33 rats), we normalized running distances by body mass of the animals (Fig. 1B, *n* = 27–29 rats). The sex differences were smaller but still significantly higher for female rats, suggesting that body mass differences can only partially explain the sex differences in running distances. Notably no significant difference was found in the running distance between the OKC-HET^B^ and OKC-HET^W^ rats within either female (Fig. 1C, *n* = 12 rats) or male rats (Fig. 1D, *n* = 12–14 rats). Our results indicate that the amount of exercise was comparable for both mitochondrial haplotypes. Overall our results demonstrate that the weekly running distance of OKC-HET rats is sex dependent but mitochondrial haplotype independent.

### Endurance exercise decreased body mass without affecting muscle mass and contractile properties

**Figure 3.**
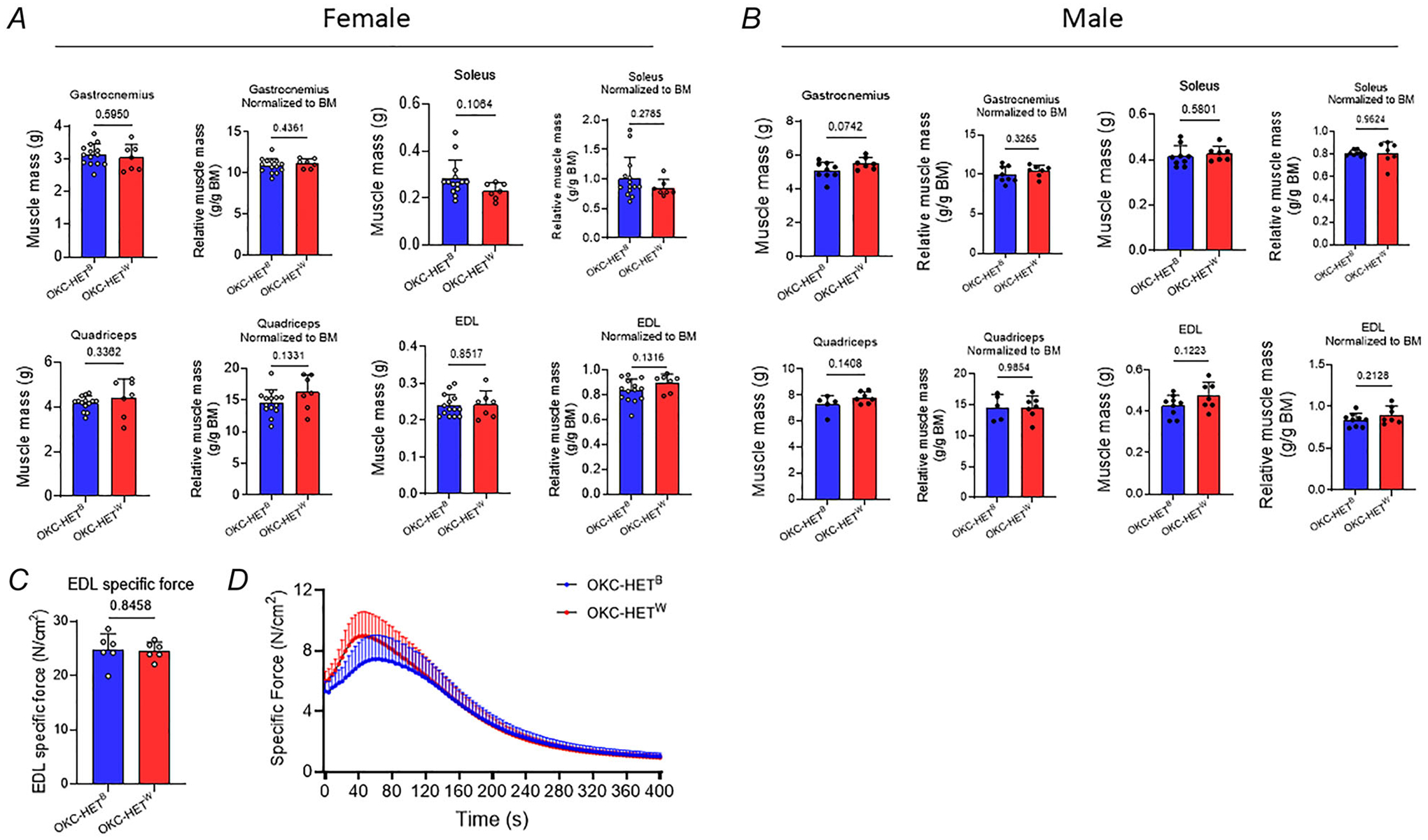
Effects of voluntary running activity on the skeletal muscle mass and force generations in OKC-HET^B/W^ rats Skeletal muscle mass of gastrocnemius, soleus, quadriceps and extensor digitorum longus (EDL) muscles, presented without (left) or with normalization to animal body mass (right) in female (*A*) and male (*B*) rats (*n* = 7–14 and *n* = 5–9, respectively). Unpaired Student’s *t* tests were used to compare means between groups. *C*, isometric maximum specific force from isolated EDL muscle in female rats. Electrical stimulation of 300 Hz was applied to induce maximum isometric specific force. *n* = 6. Unpaired *t* test. *D*, EDL was stimulated to fatigue during isometric contracts for 5 min (pulse frequency 50 Hz, train duration 500 ms and train rate 0.25 Hz) in female rats. *n* = 6. *n* indicates data from each rat. Statistical analyses were performed using a one-way ANOVA with mixed effects. Values are presented as mean ± SD.

We measured the body mass changes before and after the 8-week training period. As expected baseline average body mass was significantly higher for males than females (Fig. 2A, *n* = 20–33 rats). We observed no significant difference between the OKC-HET^B^ and OKC-HET^W^ rats for either sex (Fig. 2B,C; *n* = 10–20 rats). We found that the female rats showed a decreased body mass after the 8-week endurance training (*p* = 0.1396; Fig. 2D; *n* = 17 rats). Male rats, despite running much less than the females, lost 2.5%–5% of their baseline body mass after training (Fig. 2E, *n* = 20 rats), showing greater efficacy for training responses for male rats than females. The absence of body mass reduction following exercise training in female rats may be related to age-associated hormonal changes. At 20–21 months alterations in ovarian function and the oestrous cycle (i.e. changes in circulating oestradiol and progesterone) may attenuate the physiological responses to endurance training. Another contributing factor could be sex-specific differences in the effects of endurance exercise on food intake, as only male rats exhibited reduced food consumption, whereas female rats increased their intake (Foright et al., 2020). Genotypes did not affect body mass changes (Fig. 2F,G; *n* = 8–10 rats). We found no evidence of difference between the two mitochondrial haplotypes in muscle mass or maximum isometric specific force in isolated EDL muscle (Fig. 3A–C, *n* = 6–14 rats). Fatiguability also remained similar between the two mitochondrial haplotypes (Fig. 3D, *n* = 4–5 rats).

### Effects of mitochondrial haplotype on responses to voluntary running activities

**Figure 4.**
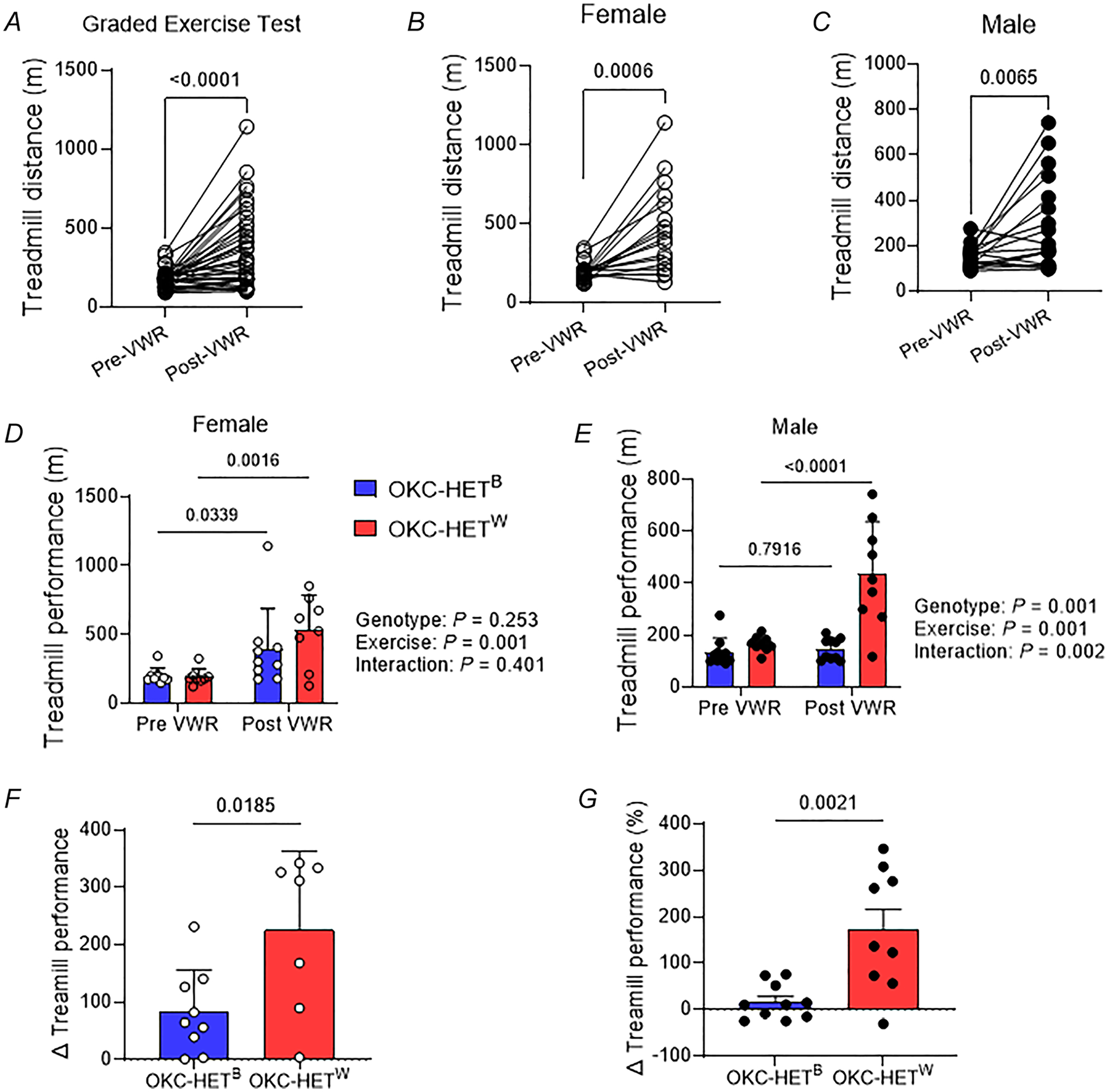
Effects of voluntary running activities on exercise tolerance in OKC-HET^B/W^ rats *A*, baseline and post-training exercise tolerance of OKC-HET rats. Exercise tolerance is measured by treadmill running distance via graded exercise protocol. *n* = 36. *B*, exercise tolerance of female rats. *n* = 17. *C*, exercise tolerance of male rats. *n* = 19. Paired *t* tests were used to compare means between groups. Treadmill running distance before and after voluntary wheel running (VWR) activities in (*D*) female and (*E*) male rats. *n* = 14–20 and 9–10, respectively. Two-way ANOVA was (genotype × exercise) used to compare means of treadmill distance between groups followed by Tukey’s *post hoc* tests. *F*, *G*, changes in treadmill running time (post–pre) were compared between genotypes in females and males. *n* = 7–9 and 9–10, respectively. *n* indicates data from each rat. Values are presented as mean ± SD.

To assess whether the mitochondrial genome influences training-induced effects on exercise capacity treadmill running times were measured before and after the 8-week exercise training. Exercise tolerance significantly increased after endurance exercise training (Fig. 4A, *n* = 36 rats). At baseline female rats ran 30% more than males (186.6 ± 8.8 m *vs*. 129.4 ± 5.9 m). After 8 weeks of VWR activities female and male rats had increased exercise tolerance by ~ 146% and 90%, respectively (Fig. 4B,C; *n* = 17–19 rats). Importantly a mitochondrial haplotype-specific effect of exercise was observed. Among females exercise tolerance increased in both OKC-HET^B^ and OKC-HET^W^ genotypes; however the increase was greater for OKC-HET^W^ than OKC-HET^B^ rats (Fig. 4D,F; *n* = 17–19 rats). Among males no changes in exercise tolerance were observed in the OKC-HET^B^ rats, but the OKC-HET^W^ rats exhibited a threefold increase (Fig. 4E,G; *n* = 17–19 rats). Thus exercise tolerance increased in OKC-HET rats, and the increases were sex- and genotype dependent.

### Effects of mitochondrial haplotype on mitochondrial bioenergetics

**Figure 5.**
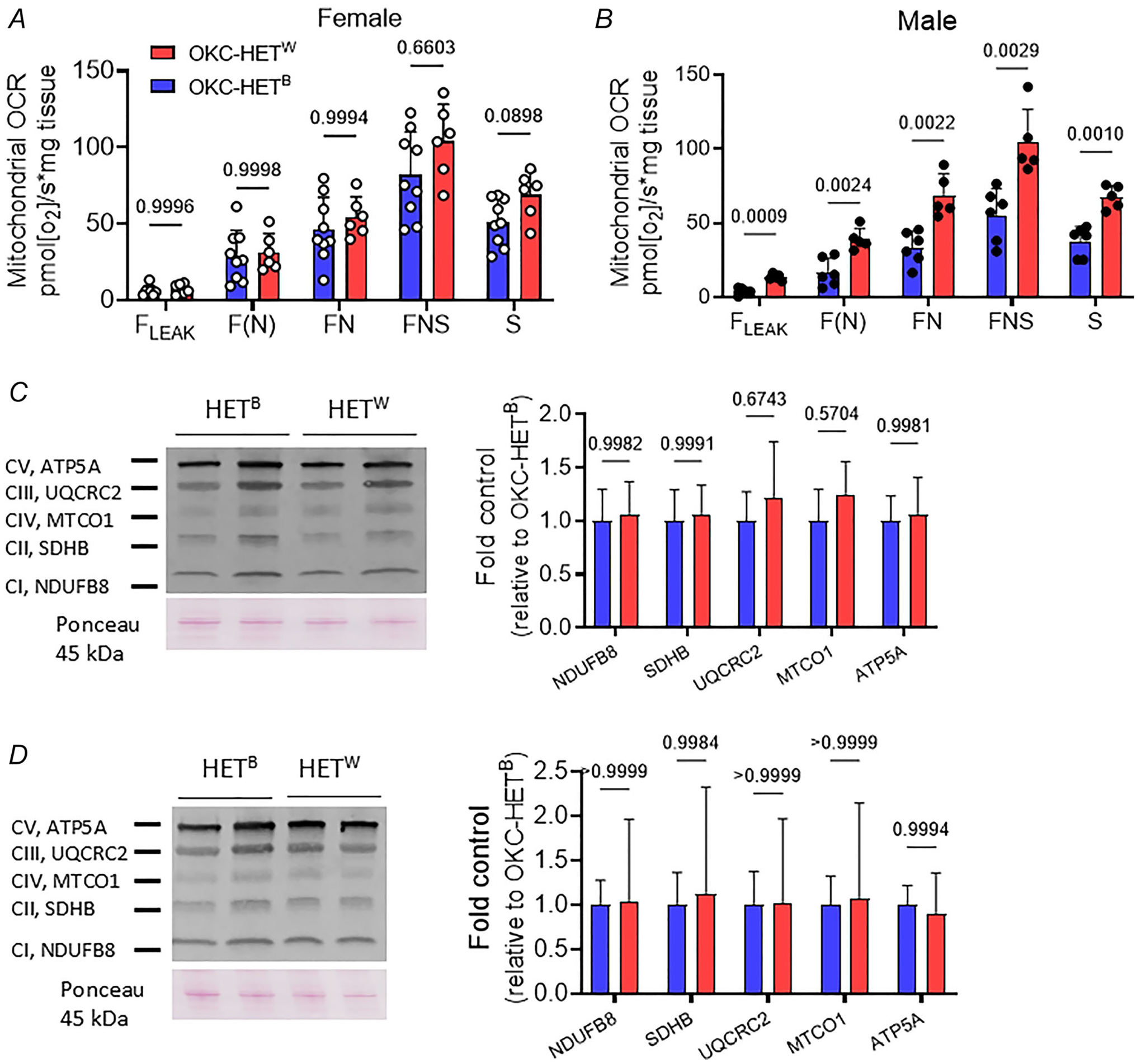
Effects of voluntary running activities on mitochondrial respiration rate of the OKC-HET^B/W^ rats Mitochondrial oxygen consumption rates of the female (*A*) and male (*B*) rats. *n* = 6–9 and 5–6, respectively. OKC-HET^B/W^ rats in response to the electron transport chain (ETC) substrates and inhibitors. *C*, expressions of representative proteins in individual mitochondrial complexes in female rats. *n* = 8. *D*, expressions of representative proteins in individual mitochondrial complexes in male rats. *n* = 8. *n* indicates data from each rat. Values are presented as mean ± SD. Abbreviation: F, fatty acid oxidation; F_LEAK_, F-linked leak respiration; F(N), F-linked Oxphos state; N, N-linked Oxphos state; FN, FN-linked Oxphos pathway; FNS, FNS-linked Oxphos pathway; S, S-linked Oxphos state.

To determine the effects of the mitochondrial haplotypes on mitochondrial bioenergetics in response to endurance exercise, we measured mitochondrial OCR using permeabilized myofibres from gastrocnemius. We sequentially added substrates that stimulate fatty acid oxidation (F)-, NADH (N)- and succinate (S)-linked pathways. Results showed that the effects of mitochondrial haplotype were dependent of sex. Female OKC-HET^W^ rats had a similar oxygen consumption rate with a trend of increase (*p* = 0.0898) in S-linked respiration (Fig. 5A, *n* = 6–9 rats). In males OKC-HET^W^ rats responded to substrates more than OKC-HET^B^ rats; mitochondrial respirations increased broadly among OKC-HET^W^ rats in the conditions we activated, including F(N)-, FN-, FNA-, S-linked respirations (Fig. 5B, *n* = 5–6 rats). To determine whether the changes in mitochondrial respiration after endurance exercise were due to changes in the components of the electron transport system (ETS), we measured levels of representative mitochondrial enzymes using whole tissue homogenates of gastrocnemius; no difference by genotype was observed (Fig. 5C,D, *n* = 8 rats). Collectively mitochondrial respiration after endurance exercise training was higher for OKC-HET^W^ than OKC-HET^B^ rats in both males and females; however this did not appear to be due to a major change in the protein expression of the ETS.

### Effects of mitochondrial haplotype on mtDNA copy number and mutation frequency

**Figure 6.**
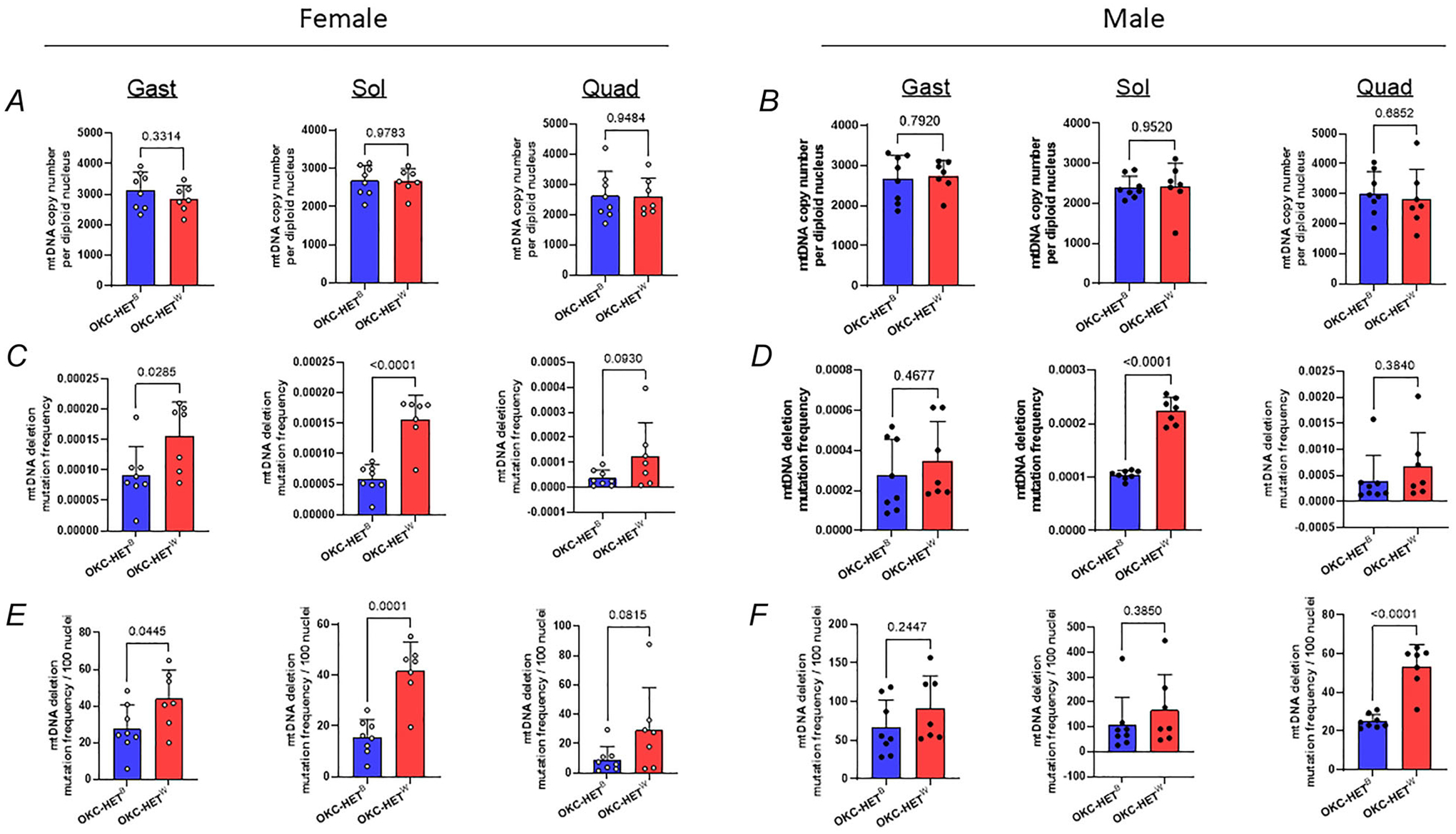
Effects of voluntary running activities on mitochondrial DNA (mtDNA) copy number and deletion mutation frequency of the male and female OKC-HET^B/W^ rats mtDNA copy number (*A*, *B)* deletion mutation frequency and (*C*, *D)* normalized mutation frequency per 100 nuclei of (*E*, *F)* female and male OKC-HET^B/W^ rats’ gastrocnemius, soleus and quadriceps muscle samples (*n* = 7–8). *n* indicates data from each rat. Students’ *t* tests were used to compare the mean of OKC-HET^B/W^ rats. Values are presented as mean ± SD. Abbreviation: Gast, gastrocnemius; Sol, soleus; Quad, quadriceps.

**Figure 7.**
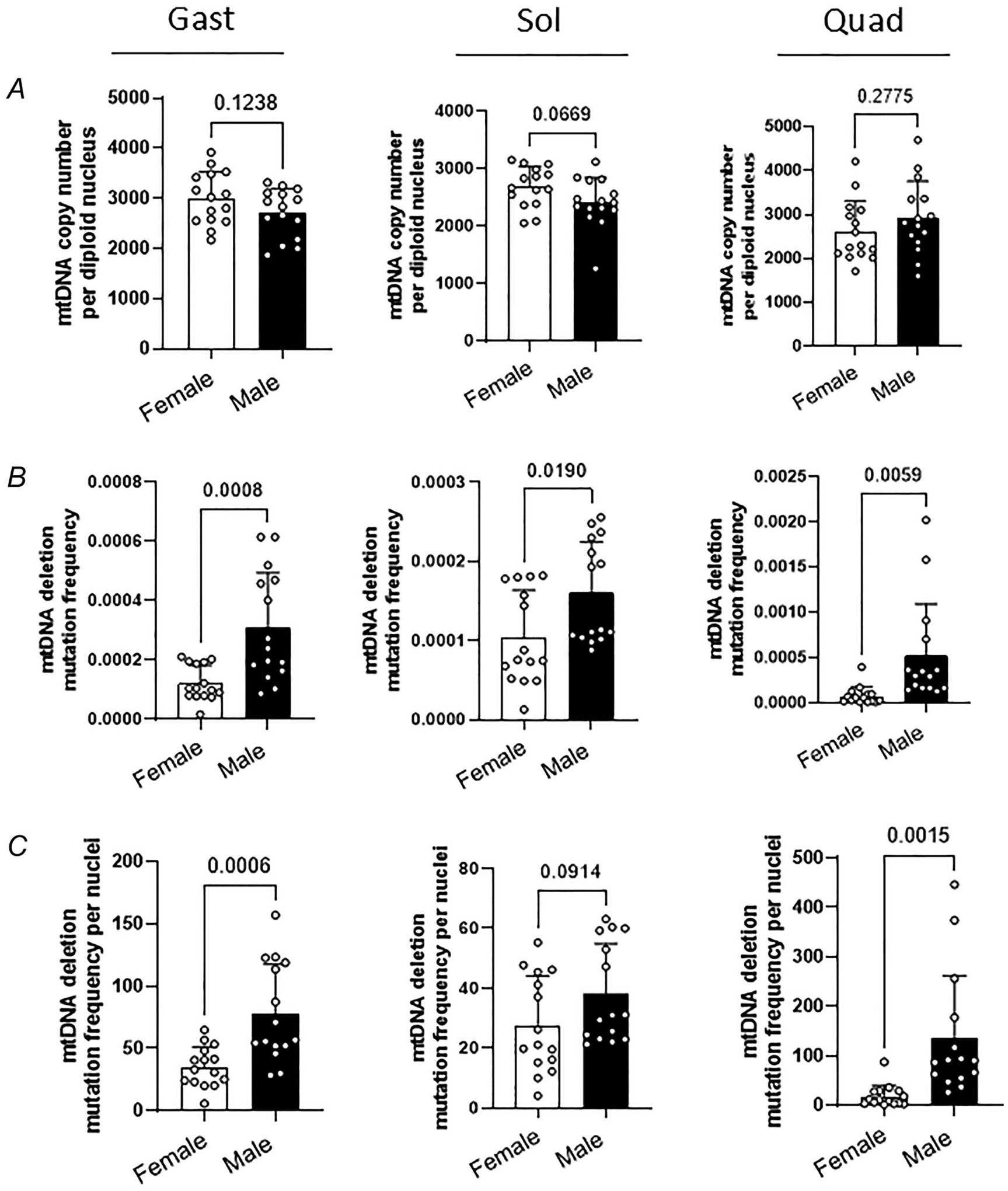
Effects of voluntary running activities on mitochondrial DNA (mtDNA) copy number and deletion mutation frequency comparing male and female OKC-HET rats *A*, mtDNA copy number deletion mutation frequency and (*B)* normalized mutation frequency per 100 nuclei of (*C)* female and male OKC-HET rats’ gastrocnemius, soleus and quadriceps muscle samples (*n* = 15). *n* indicates data from each rat. Student’s *t* tests were used to compare means of each group. Values are presented as mean ± SD. Abbreviation: Gast, gastrocnemius; Sol, soleus; Quad, quadriceps.

**Figure 8.**
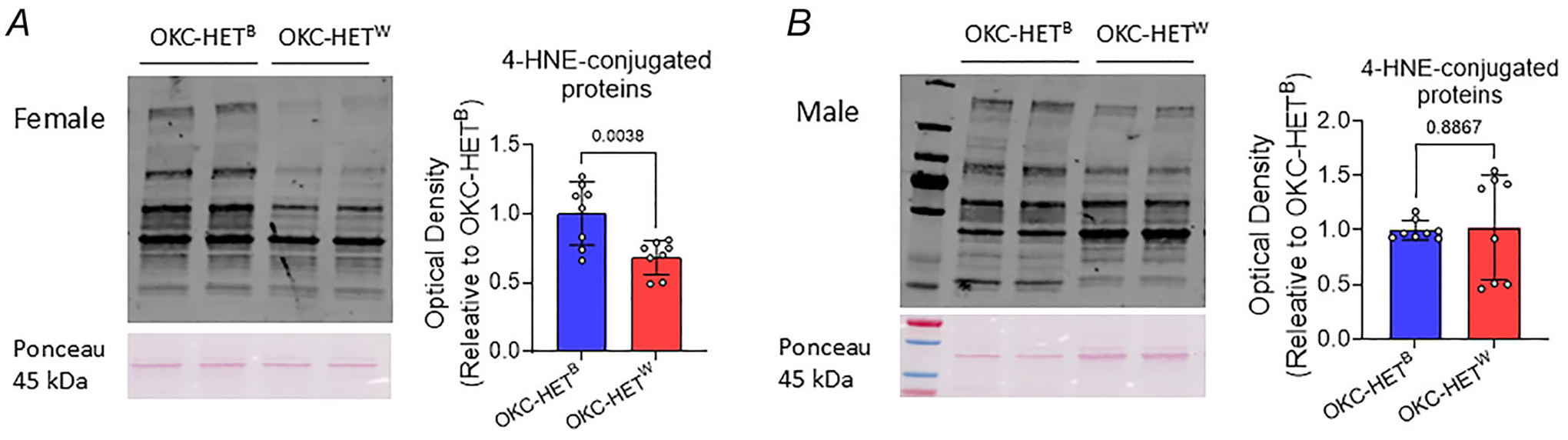
Western blot images and quantification of the 4-HNE-conjugated proteins in gastrocnemius tissue homogenates in female (*A)* and male (*B)* OKC-HET rats Optical densities are normalized to total proteins. Unpaired *t* tests were used to compare means between groups (*n* = 8). *n* indicates data from each rat. Values are presented as mean ± SD.

We measured mtDNA copy number and deletion frequency of the OKC-HET^B/W^ rats. The literature demonstrates positive correlations of mtDNA copy number with muscle size and with a marker of mitochondrial content and function (Castellani et al., 2020; McKenzie et al., 2002). The mtDNA deletion frequency exponentially increases with age, which has been associated with muscle fibre loss and accelerated mortality (Herbst et al., 2021). First we found that the mtDNA copy numbers ranged between 2400 and 3150 copies per diploid nucleus. No evidence for difference in mtDNA copy number between the two mitochondrial genotypes was observed in the gastrocnemius, soleus or quadriceps (Fig. 6A,B; *n* = 7–8 rats). However we observed significant differences in mtDNA deletion frequency between the two mitochondrial haplotypes; deletion frequency was higher for OKC-HET^W^ than OKC-HET^B^ rats for both sexes (Fig. 6C,D; *n* = 7–8 rats). Our findings were similar after normalization per 100 nuclei (Fig. 6E, F; *n* = 7–8 rats). The comparison on mtDNA copy number between male and female rats revealed no difference (Fig. 7A; *n* = 15 rats), whereas mtDNA deletion frequencies were significantly higher for males than females (Fig. 7B,C; *n* = 15 rats). To determine whether the differences in mtDNA deletions were related to changes in oxidative stress in response to endurance exercise, we measured 4-HNE, a marker of lipid peroxidation in the gastrocnemius. In females 4-HNE levels were significantly lower in OKC-HET^W^ rats than in OKC-HET^B^ rats (Fig. 8A, *n* = 8 rats). However in males we observed no difference in 4-HNE protein levels between groups (Fig. 8B, *n* = 8 rats).

### Effects of mitochondrial haplotype on motor co-ordination and neuromuscular junction

**Figure 9.**
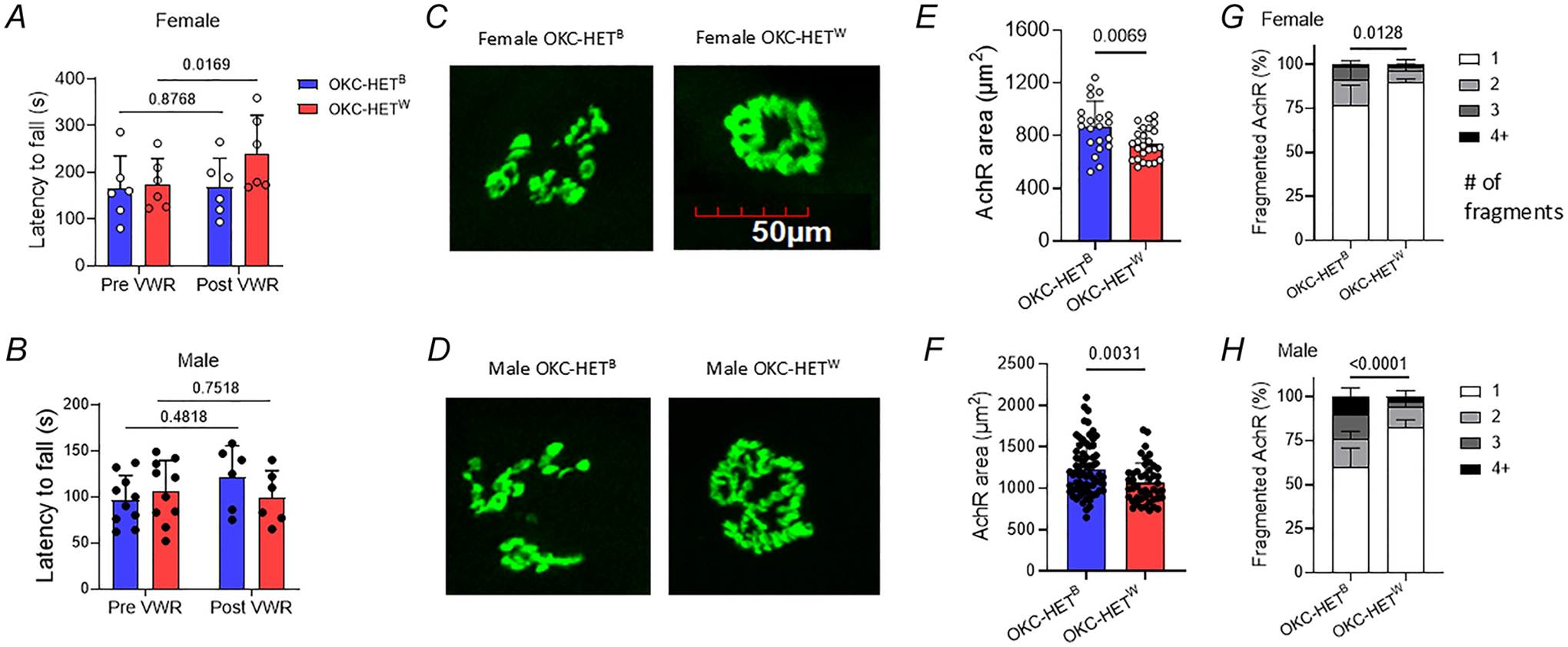
Effects of voluntary running activities on motor co-ordination and Ach receptor morphological analyses Latency to fall from the Rota Rod testing for female (*A*) and male (*B*) OKC-HET rats. *n* = 6–10. *n* indicates data from each rat. Representative images of Ach receptor (AchR) detected by bungarotoxin-*α*. Images from female (*C*) and male (*D*) OKC-HET^B^ and OKC-HET^W^ rats. Average size of AchRs for females (*E*) (*n* = 21–25) and males (*F*) (*n* = 47–70). *n* indicates individual AchRs. Percentage of fragmentated AchRs of female (*n* = 3) (*G*) and male (*n* = 5–7) (*H*) OKC-HET rats. *n* indicates individual AchRs. Values are presented as mean ± SD.

Exercise training is reported to enhance motor co-ordination and neuromuscular junction (NMJ) morphology. We used rotarod to measure motor co-ordination of OKC-HET rats after 8 weeks of voluntary exercise. In females latency to fall was ~ 40% longer in OKC-HET^W^ rats than in OKC-HET^B^ rats (Fig. 9A, *n* = 6 rats), whereas no significant differences were observed between the two mitochondrial haplotypes in male rats (Fig. 9B, *n* = 6–10 rats). Considering the role of AchRs at the NMJ and in motor function we performed immunofluorescence assays to investigate AchR structures in the gastrocnemius muscle (Fig. 9C,D). OKC-HET^W^ rats, both male and female, exhibited smaller AchR areas than OKC-HET^B^ rats (Fig. 9E,F; *n* = 21–25 and 47–70 AchRs, respectively). From AchR fragmentation analyses we found that female OKC-HET^W^ rats had fewer fragmented AchRs than OKC-HET^B^ rates (Fig. 9G, *n* = 3), whereas no significant differences were observed between the two mitochondrial haplotypes in male rats (Fig. 9H, *n* = 5–7).

## Discussion

The goal of our study was to investigate the role of mitochondrial haplotype on responses to endurance exercise training using a genetically heterogenous rat model with divergent mitochondrial genomes. Our findings reveal that female OKC-HET rats engaged in VWR at higher levels than their male counterparts, yet significant body mass loss was observed only in males. Within each sex, running activity was similar between the two strains of OKC-HET rats, demonstrating comparable exercise training. OKC-HET^W^ rats demonstrated greater responses than OKC-HET^B^ rats, showing improvements in exercise tolerance and motor co-ordination. These physiological outcomes were consistent with functional and histological data, as OKC-HET^W^ rats exhibited enhanced mitochondrial respiration and NMJ morphology compared to OKC-HET^B^ rats. The mitochondrial haplotype effects on training effects and mtDNA mutation frequencies were dependent on the sex of the animals. Together our data show that mitochondrial genome and sex directly impact the responses to endurance training in genetically heterogenous rats.

Endurance exercise in rodents is commonly conducted using either running wheels or treadmills. In this study we selected running wheels to investigate training effects because they allow for voluntary activity in contrast to forced exercise imposed by treadmill running that have an impact on lifespan (Narath et al., 2001). Our approach more accurately reflects the voluntary nature of human exercise. We observed that the voluntary running activities of male OKC-HET rats were similar to those of rats aged 20–22 months in previous reports (Ko & Ko, 2021; Scarpace et al., 2012). Notably prior studies predominantly used male rats, although only a few studies used females (Fuller et al., 2006; Hyatt et al., 2019; Ishihara et al., 1991; Kariya et al., 2004). Comparing voluntary running distances between sexes, we found that female OKC-HET rats ran significantly more than males, with females running approximately five times more than males at baseline and eight times more by week 8. Normalizing running distance by body mass revealed that body mass only partially accounted for these differences. Consistent with previous studies we observed that female rodents run significantly more than males (De Bono et al., 2006; Jones et al., 1990; Rosenfeld, 2017), with sex differences influenced by age (Holloszy et al., 1985), oestrogen and oestrogen receptors (Gorzek et al., 2007; Ogawa et al., 2003). Within each sex running activities and the effects of training on body mass were comparable between OKC-HET^B^ and OKC-HET^W^ rats, indicating similar exercise training across mitochondrial haplotypes.

Numerous groups have investigated how skeletal muscle responses to endurance training and the reports consistently demonstrate that endurance training enhances mitochondrial density, biogenesis and mitophagy (Morton et al., 2019; Smuder et al., 2019), which lead to improved bioenergetics and reduced ROS production. Exercise has also been shown to increase insulin sensitivity in models of diet-induced obesity (Bradley et al., 2008) and to protect against age-related neuromuscular impairments, such as AchR fragmentation and NMJ disruption (Valdez et al., 2010). Additionally endurance exercise increases the expression of antioxidant enzymes (Morton et al., 2019), which play a crucial role in mediating the protective effects of exercise. These findings in animal models are generally consistent; however human responses to exercise are far more variable (Erickson et al., 2023). Some individuals respond robustly to endurance exercise; others exhibit minimal acclimation. A major contributor underlying this variability is genetic diversity (Sarzynski et al., 2022). Humans possess significant genetic variation, whereas most of the laboratory mice used are inbred lines that are genetically homogeneous. This difference presents a critical barrier to progress in exercise physiology and muscle biology, as insights from genetically identical animal models may not fully translate to the genetically diverse human population (Sathiaseelan et al., 2023).

The OKC-HET^W^ rats exhibited higher mtDNA mutation frequencies than OKC-HET^B^ rats. Vandiver et al. reported that mtDNA deletion mutation frequencies in skeletal muscle exponentially increase with age, which was associated with muscle fibre loss and accelerated mortality (Vandiver et al., 2023). Increased mtDNA deletion mutations in OKC-HET^W^ rats were unexpected and intriguing (Fig. 5). One possibility is that exercise-induced mitochondrial reactive oxygen species (mtROS) signalling may be more pronounced in the OKC-HET^W^ rats, resulting in oxidative modifications within the mitochondria that increase deletion mutations. In this scenario mtROS could exert both beneficial and adverse effects (Powers et al., 2024), activating signalling pathways via protein modifications in the cytoplasm while simultaneously elevating mtDNA deletion mutations. Our results indicate that the increased mtDNA deletion mutations did not lead to any morphological or functional issues after the training period. Whether the levels of deletion mutations observed in OKC-HET^W^ rats will be deleterious later in life remains undetermined. Despite the higher frequencies of deletion mutations in mtDNA, markers of lipid peroxidation in whole tissue homogenate were lower in OKC-HET^W^ rats than OKC-HET^B^ in female rats. The mtDNA deletion mutations may influence differential training responses to endurance exercise in OKC-HET rats, as mtDNA mutations have been shown to alter responses to endurance exercise (Schaefer et al., 2022).

Endurance training has been shown to impact mtDNA deletion mutations in rodents. Male Sprague–Dawley rats trained on a treadmill for 12 weeks showed an increased frequency of mtDNA deletions in the myocardium (Huang et al., 2009). Another group trained adult male rats on a treadmill at moderate and high intensities for 12 weeks and measured mtDNA deletion mutations in soleus muscle with predominant slow twitch myofibres. The rats with high-intensity training exhibited increased mtDNA deletion, whereas the rats with moderate intensity did not show increases in mtDNA deletion (Jafari et al., 2005). Similar to the training effect an acute aerobic exercise at a running speed of 40 m/min for 20 min increased mtDNA 380 bp deletion in soleus muscle of adult male rats (Sakai et al., 1999). Studies have shown that mtDNA deletion mutations in muscle increase after exercise training. Notably to our knowledge our research is the first to demonstrate the effects of mitochondrial haplotype on mtDNA deletion mutations in response to exercise.

The literature reports mixed effects of endurance exercise training on mtDNA copy number. In male C57Bl/6 mice increased mtDNA copy number was detected in mice subject to 20 week swimming exercise relative to sedentary animals (Cao et al., 2012). Another study found no changes in mtDNA copy number in plantaris muscle of male Wistar rats after 6 weeks of treadmill running (Koma et al., 2024). In a pilot study involving eight patients (both women and men) with heteroplasmic mtDNA mutations, 14 weeks of moderate-intensity cycling training did not affect mtDNA copy number (Schaefer et al., 2022). In OKC-HET rats mitochondrial genotype did not affect mtDNA copy number in response to exercise in both male and female animals.

Mitochondrial genome affects the NMJ disruption and motor co-ordination after endurance training. In line with the previous findings (Valdez et al., 2010) our results show that female OKC-HET rats displayed enhanced motor co-ordination after 8 weeks of voluntary running activities. Morphological analysis of AchRs revealed that female OKC-HET^W^ rats had less-fragmented AchRs than OKC-HET^B^ rats. Unlike female OKC-HET^W^ rats male rats did not demonstrate increased motor co-ordination; however fragmentation was reduced in both male and female OKC-HET^W^ rats. The mitochondrial haplotype has significant effects on motor co-ordination and AchR structure in a sex-specific manner. Differences in sex hormones and training amount might drive the sex differences, warranting future studies. Another area for future research is the effect of endurance exercise on the motor neurons, which are upstream of NMJ disruption. In male Sprague–Dawley rats 50% of the motor neurons show age-related cell death that occurs before NMJ disruption and AchR disintegrations (Cowen et al., 2000).

Biological sex significantly influences how the mitochondrial genome affects acclimation responses to endurance exercise. In female rats treadmill running distance post-training increased for both haplotypes, whereas in male rats this increase was observed only for OKC-HET^W^ rats. In skeletal muscle mitochondria, differences in OCR were apparent across all respiratory states in males but were largely similar in females. The mtDNA deletions were detected in the soleus muscle of both sexes but were present only in the gastrocnemius muscle of females. Markers of lipid peroxidation varied between OKC-HET^B^ and OKC-HET^W^ rats in females but not in males. Additionally mtDNA deletion frequencies were significantly higher in males compared to females. The factors contributing to these sex differences remain unclear and are beyond the scope of this study.

The interaction between mitochondrial and nuclear genomes represents a crucial but often overlooked aspect of cellular function. Mitochondrial activity relies on over 1200 proteins synthesized in the nucleus, with only 13 proteins encoded by the mitochondrial genome. These 13 proteins play vital roles in metabolism, forming key components of four of the five complexes in the ETS. Consequently precise co-ordination of transcription, translation and transport of these mitonuclear proteins is essential across various cellular conditions, especially those influencing mitochondrial biogenesis or mitophagy (Quirós et al., 2016). Another distinct feature of mitonuclear interactions stems from the fact that mitochondria are inherited exclusively through the female lineage. This inheritance pattern exerts stronger selective pressure on optimizing mitonuclear co-ordination in females; any male-specific mutations in mtDNA tend to have limited evolutionary impact. This sex-specific inheritance pattern has been suggested to contribute to differences in longevity and age-related diseases between males and females (Frank, 2012). In studies on *Drosophila* mitochondrial haplotype has been shown to influence ageing phenotypes in a sex-specific manner (Camus et al., 2012) consistent with our data.

A limitation of our study is the significant sex differences in voluntary activities. Male rats show the greater increases in body mass and fat with increasing age than females (Narath et al., 2001; Turturro et al., 1999), which likely contributes to the differences in the voluntary activities between sexes. Although our study used older animals with focuses on the effects of endurance training on sarcopenia, younger animals generally exhibit higher activities than older ones (Narath et al., 2001) and expected to exhibit more similar voluntary activities across sexes than older animals.

In summary our data show for the first time that the mitochondrial haplotype significantly impacts training responses to endurance exercise in a sex-specific manner. Using OKC-HET^B/W^ rats we demonstrated that exercise-induced changes are mitochondrial haplotype-specific, with some phenotypes manifesting in one sex but not the other. Our previous work identified 94 nucleotide differences between the two mitochondrial genomes, including 16 non-synonymous substitutions in the genes for ND2, COI, ATPase8, ATPase6, ND4, ND6 and Cytb (Sathiaseelan et al., 2023). OKC-HET rats may serve as a model for testing the functional significance of specific mtDNA variants in endurance training responses.

## Supplementary Material

Supplementary Material

The peer review history is available in the Supporting Information section of this article (https://doi.org/10.1113/JP288330#support-information-section).

Additional supporting information can be found online in the Supporting Information section at the end of the HTML view of the article. Supporting information files available:

Peer Review History

## Data Availability

All the data presented in the manuscript can be provided upon reasonable requests to the corresponding author.

## Key points

- Mitochondrial haplotype affects the responses to endurance exercise.
- Sex modulates the effects of mitochondrial haplotype in the responses to endurance training.
- Mitochondrial DNA (mtDNA) deletion frequency increases following endurance exercise.
- mtDNA deletion frequency is higher in males than females after endurance exercise in OKC-HET rats.
