# Supplementary material for: Mitochondrial haplotype and sex modulate responses to endurance exercise training
Source: J Physiol. Author manuscript; Available in PMC 2026 Apr 29. (PMC13127427; doi:10.1113/JP288330)
Supplement: Supplementary Material [file NIHMS2161071-supplement-Supplementary_Material.pdf]

The Journal of Physiology

Mitochondrial Haplotype and Sex Modulate Responses to Endurance Exercise Training

Bumsoo Ahn, Tianhao Wei, Ryan Pettit-Mee, Eunyoung Kim, Robert Vincent Musci, Jonathan Wanagat, Hoang Van Nguyen, Arlan Richardson, and Hyunyoung Kim
DOI: 10.1113/JP288330

Corresponding author(s): Bumsoo Ahn (bahn@wakehealth.edu)

The following individual(s) involved in review of this submission have agreed to reveal their identity: Erich Gnaiger (Referee #1); James P DeLany (Referee #2)

Review Timeline:

Submission Date:	17-Dec-2024
Editorial Decision:	17-Feb-2025
Revision Received:	13-Mar-2025
Editorial Decision:	09-Apr-2025
Revision Received:	13-Apr-2025
Accepted:	30-Apr-2025

Senior Editor: Paul Greenhaff

Reviewing Editor: Russell Hepple

Transaction Report:

(Note: With the exception of the correction of typographical or spelling errors that could be a source of ambiguity, letters and reports are not edited. Depending on transfer agreements, referee reports obtained elsewhere may or may not be included in this compilation. Referee reports are anonymous unless the Referee chooses to sign their reports.)

1st Editorial Decision 17-Feb-2025

Dear Dr Ahn,

Re: JP-RP-2024-288330 "Mitochondrial Haplotype and Sex Modulate Adaptive Responses to Endurance Exercise Training" by Bumsoo Ahn, Tianhao Wei, Ryan Pettit-Mee, Eunyoung Kim, Robert Vincent Musci, Jonathan Wanagat, Hoang Van Nguyen, Arlan Richardson, and Hyunyoung Kim

Thank you for submitting your manuscript to The Journal of Physiology. It has been assessed by a Reviewing Editor and by 2 expert referees and we are pleased to tell you that it is acceptable for publication following satisfactory revision.

The referee reports are copied at the end of this email.

Please address all the points raised and incorporate all requested revisions or explain in your Response to Referees why a change has not been made. We hope you will find the comments helpful and that you will be able to return your revised manuscript within 4 weeks. If you require longer than this, please contact journal staff: jp@physoc.org.

Your revised manuscript should be submitted online using the link in your Author Tasks Link Not Available. This link is accessible via your account as Corresponding Author; it is not available to your co-authors. If this presents a problem, please contact journal staff (jp@physoc.org). Image files from the previous version are retained on the system. Please ensure you replace or remove any files that are being revised.

If you do not wish to submit a revised version of your manuscript, you must inform our journal staff (jp@physoc.org) or reply to this email to request withdrawal. Please note that a manuscript must be formally withdrawn from the peer review process at one journal before it may be submitted to another journal.

TRANSPARENT PEER REVIEW POLICY: To improve the transparency of its peer review process The Journal of Physiology publishes online, as supporting information, the peer review history of all articles accepted for publication. Readers will have access to decision letters, including Editors' comments and referee reports, for each version of the manuscript, as well as any author responses to peer review comments. Referees can decide whether or not they wish to be named on the peer review history document.

ABSTRACT FIGURES: Authors are expected to use The Journal's premium BioRender account to create/redraw their Abstract Figures. Information on how to access this account is here: https://physoc.onlinelibrary.wiley.com/journal/14697793/biorender-access.

This will enable Authors to create and download high-resolution figures. If authors have used the free BioRender service, they can use the instructions provided in the link above to download a high-resolution version suitable for publication.
The link provided should only be used for the purposes of this submission. Authors will be charged for figures created on this account if they are not related to this manuscript submission.

LANGUAGE EDITING AND SUPPORT FOR PUBLICATION: If you would like help with English language editing, or other article preparation support, Wiley Editing Services offers expert help, including English Language Editing, as well as translation, manuscript formatting, and figure formatting at www.wileyauthors.com/eeo/preparation. You can also find resources for Preparing Your Article for general guidance about writing and preparing your manuscript at www.wileyauthors.com/eeo/prepresources.

RIGOUR AND REPRODUCIBILITY: We recommend authors consult The Journal's Rigour and Reproducibility requirements to ensure that their manuscript is suitable for publication: https://physoc.onlinelibrary.wiley.com/pb-assets/hub-assets/physoc/documents/TJP-Rigour-and-Reproducibility-Requirements.pdf

REVISION CHECKLIST:
Check that your Methods section conforms to journal policy: https://jp.msubmit.net/cgi-bin/main.plex?form_type=display_requirements#methods

Check that data presented conforms to the statistics policy: https://jp.msubmit.net/cgi-bin/main.plex?form_type=display_requirements#statistics

Upload a full Response to Referees file. To create your 'Response to Referees' copy all the reports, including any comments from the Senior and Reviewing Editors, into a Microsoft Word, or similar, file and respond to each point, using font or background colour to distinguish comments and responses and upload as the required file type.

Please upload two versions of your manuscript text: one with all relevant changes highlighted and one clean version with no changes tracked. The manuscript file should include all tables and figure legends, but each figure/graph should be uploaded as separate, high-resolution files. The journal is now integrated with Wiley's Image Checking service. For further details, see: https://www.wiley.com/en-us/network/publishing/research-publishing/trending-stories/upholding-image-integrity-wileys-image-screening-service

You may also upload:
- 'Potential Cover Art' for consideration as the issue's cover image
- Appropriate Supporting Information (Video, audio or data set: see https://jp.msubmit.net/cgi-bin/main.plex?form_type=display_requirements#supp).

We look forward to receiving your revised submission.

If you have any queries, please reply to this email and we will be pleased to advise.

Yours sincerely,

Paul Greenhaff
Senior Editor
The Journal of Physiology

REQUIRED ITEMS

- Author photo and profile. First or joint first authors are asked to provide a short biography (no more than 100 words for one author or 150 words in total for joint first authors) and a portrait photograph. These should be uploaded and clearly labelled together in a Word document with the revised version of the manuscript. See Information for Authors for further details.

- You must start the Methods section with a paragraph headed Ethical approval (https://jp.msubmit.net/cgi-bin/main.plex?form_type=display_requirements#methods).

Research must comply with The Journal's policies regarding animal experiments (https://physoc.onlinelibrary.wiley.com/hub/animal-experiments) and adherence to these policies must be stated in the manuscript.

Authors should confirm in their Methods section that their experiments were carried out according to the guidelines laid down by their institution's animal welfare committee, including an ethics approval reference number. The Methods section must contain a statement about access to food, water and housing, details of the anaesthetic regime: anaesthetic used, dose and route of administration, and method of killing the experimental animals.

- Your manuscript must include a complete Additional Information section, including competing interests; funding; author contributions and acknowledgements.

- Please upload separate high-quality figure files via the submission form.

- You must upload original, uncropped western blot/gel images (including controls) if they are not included in the manuscript. This is to confirm that no inappropriate, unethical or misleading image manipulation has occurred. These should be uploaded as 'Supporting information for review process only'. Please label/highlight the original gels so that we can clearly see which sections/lanes have been used in the manuscript figures. For more information, see: https://physoc.onlinelibrary.wiley.com/hub/journal-policies#imagmanip.

- Please ensure that the Article File you upload is a Word file.

- Papers must comply with the Statistics Policy: https://jp.msubmit.net/cgi-bin/main.plex?form_type=display_requirements#statistics.

In summary:

- If n {less than or equal to} 30, all data points must be plotted in the figure in a way that reveals their range and distribution. A bar graph with data points overlaid, a box and whisker plot or a violin plot (preferably with data points included) are acceptable formats.

- If n > 30, then the entire raw dataset must be made available either as supporting information, or hosted on a not-for-profit repository, e.g. FigShare, with access details provided in the manuscript.

- 'n' clearly defined (e.g. x cells from y slices in z animals) in the Methods. Authors should be mindful of pseudoreplication.

- All relevant 'n' values must be clearly stated in the main text, figures and tables.

- The most appropriate summary statistic (e.g. mean or median and standard deviation) must be used. Standard Error of the Mean (SEM) alone is not permitted.

- Exact p values must be stated. Authors must not use 'greater than' or 'less than'. Exact p values must be stated to three significant figures even when 'no statistical significance' is claimed.

- Please include an Abstract Figure file, as well as the Figure Legend text within the main article file. The Abstract Figure is a piece of artwork designed to give readers an immediate understanding of the research and should summarise the main conclusions. If possible, the image should be easily 'readable' from left to right or top to bottom. It should show the physiological relevance of the manuscript so readers can assess the importance and content of its findings. Abstract Figures should not merely recapitulate other figures in the manuscript. Please try to keep the diagram as simple as possible and without superfluous information that may distract from the main conclusion(s). Abstract Figures must be provided by authors no later than the revised manuscript stage and should be uploaded as a separate file during online submission labelled as File Type 'Abstract Figure'. Please also ensure that you include the figure legend in the main article file. All Abstract Figures should be created using BioRender. Authors should use The Journal's premium BioRender account to export high-resolution images. Details on how to use and access the premium account are included as part of this email.

- Please include a full title page as part of your main article (Word) file, which should contain the following: title, authors, affiliations, corresponding author name and contact details, keywords, and running title.

- Please ensure that all figures and tables have a title and legend, and that they have been cited within the main article text.

EDITOR COMMENTS

Reviewing Editor:

Thank you for submitting your work to The Journal of Physiology. Whereas both reviewers were enthusiastic about the novelty of the work, both had comments that will need to be addressed in a revision. Reviewer 1 was primarily concerned with accuracy in terminology and makes some good points concerning the mitochondrial substrate-inhibitor protocol that was employed. Reviewer 2's main request was additional information about what is encoded by the 94 nucleotides that differ between the two rat strains. This should be relatively easy to determine and should be provided by the authors in their revision. An additional question concerns the rationale for studying 18-19 mo old animals. Are the survival characteristics of the rats with the different mitochondrial haplotypes known and could differences in the % survival between them contribute to the differences between strains that were observed. For example, if one strain were biologically older (at a lower %survival) despite being the same absolute age (in months), this could presumably affect the amount of voluntary activity and the nature of the muscle alterations. Further to this point, since male rats tend to show a considerably greater increase in body fat with increasing age than females (see Turturro et al. J Gerontol Biol Sci Med Sci 54[11]: B492-501, 1999), this is a likely contributor to the differences in voluntary activity between male and female rats and may not have been as extreme at younger ages. This point should be addressed by the authors in their revision.

Senior Editor:

Comments for Authors to ensure the paper complies with the Statistics Policy:
Exact p values not used, data shown as mean +/- SEM, not SD.

Comments to the Author:
Thank you for the Manuscript submission to The Journal of Physiology. It has been considered by a reviewing editor and two expert reviewers. All were positive about the work but have raised a number of important points that need to be considered by the authors. In addition, in the "study design" section of the Methods please state the total number of animals used. This is important because of the different animal numbers used in study groups depicted in the Figures. Please also ensure the manuscript complies to the Journal statistics policy, i.e. please use exact p values and present data as mean +/- SD, not SEM throughout. We look forward to receiving the revised manuscript. Thank you for considering The Journal of Physiology.

REFEREE COMMENTS

Referee #1:

JP-RP-2024-288330 J Physiol: Mitochondrial Haplotype and Sex Modulate Adaptive Responses to Endurance Exercise Training

This manuscript highlights the relevance of mt genetic diversity and biological sex for understanding the variability of physiological responses to endurance training using OKC-$HET^{B/W}$ rats. Exercise tolerance increased to a greater extent in OKC-HET^{W} than OKC-HET^{B} rats. This is a fundamental contribution to evaluate the use of animal models for extrapolation to humans.

Comments (major and minor):

'Exercise adaptation' (Abstract) or 'adaptation to endurance exercise' (Introduction) is a widely and ambiguously used term and should be re-considered in an evolutionary and comparative physiological context. It is useful to restrict the term 'adaptation' to an evolutionary time scale, in contrast to 'exercise acclimation' studied at an immediate time scale; ref Hochachka PW, Somero GN (2002) Biochemical adaptation: mechanism and process in physiological evolution. Oxford Univ Press, New York: 466 pp. If 'acclimation' is defined as an immediate time scale adaption expressing phenotypic plasticity in response to changes of a single variable under controlled laboratory conditions, then 'adaption' may be considered as the general term, perhaps less technically compared to acclimation or even acclimatization. Following a concept of rigorously defined terms is particularly helpful in the present context when 'a large portion of exercise response variability is attributable to genetic heritability'.

p. 3: 'maximum oxygen consumption rate (OCR)' - as used here, is this equivalent to V_{O2max}, or what is the difference?

p. 3: 'mitochondria are .. responsible for producing most of the cellular energy' and 'energy production' (Introduction) - The concept of 'energy production' contradicts the First Law of thermodynamics. I recommend the substitute 'transforming' for 'producing'.

p. 4: 'Maximal OCR of humans (Bassett & Howley, 2000) is significantly closer to that of rats (Bedford et al., 1979; Rodrigues et al., 2007) than of mice (Fernando et al., 1993; Schefer & Talan, 1996).' - It is necessary to give the units of the measure of OCR and refer to the underlying concept of scaling, to clarify the basis of comparing OCR in humans, rats, and mice. Is the OCR of humans closer to that of rats on the respiratory physiology or mitochondrial level, or both?

p. 5, 9, 11, and several figures: 'weight' - must be replaced by 'mass'. The Bureau International des Poids et Mesures (2019) The International System of Units (SI). 9th edition:117-216 declares 'the necessity to put an end to the ambiguity which in current practice still exists on the meaning of the word *weight*, used sometimes for *mass*, sometimes for *mechanical force* .. The word "weight" denotes a quantity of the same nature as a "force": the weight of a body is the product of its mass and the acceleration due to gravity'.

p. 6: 'rats were sacrificed by carbon dioxide' - this may need a more detailed description.

Typo p. 7: '10mM Ca-EGTA buffer - insert space: 10 mM

p. 7: 'Van Remmen et al., 2019' - The reference 'Van Remmen H, Pharaoh G & Ahn B. (2019). The Role of Mitochondrial Peroxide Release in the Mechanisms Underlaying Age-Related Sarcopenia. Faseb Journal 33.' (p. 22) is incomplete and could not be found in PubMed.

p. 7: 'previously described method (Ahn et al., 2019 ..) - The protocol described in this reference is entirely different from the protocol used in the present manuscript. 'Rates of respiration were determined using sequential additions of substrates and inhibitors as follows: octanoylcarnitine (0.5 mM), malate (0.1 mM), ADP (2.5 mM), glutamate (10 mM), malate (2 mM), succinate (10 mM), rotenone (0.5 µM), Antimycin A (2.5 µM), and TMPD (0.5 mM) immediately followed by ascorbate (5 mM).' The rationale for this protocol is not explained, and references to the original publication(s) describing essential sections of this protocol are missing. Why is malate used sequentially at 0.1 mM and 2 mM? See https://doi.org/10.1152/ajpendo.00475.2015 and https://doi.org/10.1016/j.bbadis.2024.167544. Why is cytochrome c not added, which may be required for full CIV activity? How was oxygen consumption after addition of TMPD (0.5 mM) and ascorbate (5 mM) corrected for autoxidation of ascorbate and TMPD? If no corrections were applied, the 'CIV' data are artefacts and should be excluded from the results section.

p. 7: 'All respiration measurements were normalized to Antimycin A to account for non-mitochondrial oxygen consumption.' - The term 'normalization' is confused with 'baseline correction'. 'Non-mitochondrial oxygen consumption' suggests that inhibition of the electron transfer system by Antimycin A inhibits all other oxygen-consuming processes in mitochondria. To correct for these ambiguities, the sentence should be written as: 'All respiratory rates were corrected for residual oxygen consumption measured after inhibition of the mitochondrial electron transfer system by Antimycin A.'

p. 7: While mitochondrial respiration was measured at 37 {degree sign}C, measurement of force-frequency EDL muscle was performed at 32 {degree sign}C. The rationale for this difference should be explained.

p. 8: '*$p < .05$'. See: Amrhein V, Greenland S, McShane B (2019) Scientists rise up against statistical significance. Nature 567:305-307.

p. 9: 'no significant difference was found in the running distance between the OKC-HET^{B} and OKC-HET^{W} rats within .. male (Fig. 1D) rats.' - Is the apparent (small) difference between the male haplotypes at weeks 1 to 5, and it's disappearance at weeks 7 to 8 of any interest?

p. 10: 'We sequentially added substrates that activate fatty acid oxidation, complex I, complex II, and complex IV.' - For the non-specialist, this terminology may be misleading. After addition of substrates (see p. 7) glutamate (10 mM) and malate (2 mM), the following respiratory Complexes are stimulated: CI, CIII, and CIV. After further addition of succinate and rotenone, the following respiratory Complexes are stimulated: CII, CIII, and CIV. 'Activation' in enzyme kinetics is to be distinguished from 'stimulation' by simply adding a substrate or, on the contrary, an inhibitor.

Figure 5 A and B: The coupling state LEAK requires further explanation in terms of pathway state. In the present SUIT protocol, it is the state (rate) FAO_L (or F_L). After addition of ADP (2.5 mM), the respiratory state is not 'State 3', but OXPHOS capacity at saturating ADP concentration (https://doi.org/10.26124/bec:2020-0001.v1). Compared to FAO (the pathway of fatty acid oxidation; strictly acylcarnitine oxidation when octanoylcarnitine plus malate 0.1 mM are used), C-1 is misleading (it is not CI activity) and is more correctly abbreviated as CI-linked or in terms of substrate&inhibitor-defined pathway capacity simply abbreviated as N (with NADH-linked substrates), and then NS (instead of C-I&II). C-I(Rot) is wrong: After

inhibition of CI by rotenone, the pathway capacity is CII-linked (not C-I), simply indicated as S (succinate). CIV is in the present protocol CIV-OXPHOS capacity (if corrected for O2 concentration-dependent autoxidation of ascorbate and TMPD, and if not limited by cytochrome c). See https://doi.org/10.26124/bec:2020-0002 .

Typo p. 10: 'no significant differences were observed between the two mt haplotypes in male rats (Fig. 8B). - This should be Fig. 9B.

Figure 9F: The data look very different from $^{**}P < .01$. It is more convincing to give the actual *P*-value (see reference suggested for p. 8).

p. 11: 'Consistent with previous studies, we observed that female rodents run significantly more than males (Jones et al., 1990; Rosenfeld, 2017)' - The references could be extended to include more results on mice: https://doi.org/10.1152/ajpregu.00694.2005, https://doi.org/10.1590/1414-431X20187830

Referee #2:

This manuscript addresses a very important characteristic when using rodent models in an attempt to understand the role of genetics in exercise response variability, that many investigators may not be aware of. That is, most investigations using rodent models use inbred strains, which have no mitochondrial diversity, because each inbred strain of laboratory mice originates from a single female mouse, and hence each mouse studied has the same mitochondrial genetics. The investigators utilized a novel OKC-HET rat model, which has 94 nucleotides mtDNA differences between the OKCHETB and OKC-HETW rats, to investigate the impact of mt haplotype on exercise training (voluntary wheel running).

One of the most striking findings was that females engaged in approximately 5-8 times as much voluntary wheel running than males. However, wheel running distance was not affected by mtDNA differences.

Exercise tolerance increased in both male and female rats in response to endurance exercise training. There was a significant effect of mtDNA haplogroup, with the increased endurance being greater in the OKC-HETw female rats, and only apparent in males with the OKC-HETw mtDNA haplogroup. Consistent with the greater response in the OKC-HETw rats, mitochondrial respiration was higher in the OKC-HETw rats. However, neither the expression of representative proteins in individual mitochondrial complexes nor mtDNA copy number played a role in these mtDNA haplogroup differences since these were not different by mtDNA haplogroup.

One parameter usually associated with negative health outcomes, mtDNA deletion frequency was higher in the OKC-HETw rats. However, 4-HNE, a marker of lipid peroxidation was lower in the female OKC-HETw rats.

Specific Comments

1) Regarding the strikingly higher wheel running in the female rats:

a. Do the authors think that the outcomes would be different if they instituted a similar exercise training level in the males and females? And would that have been a better approach?

b. How do the authors reconcile the body weight loss after exercise, with no weight loss in females, but significant weight loss in males?

c. In light of the extreme sex difference in exercise training and lack of concordant weight loss, how do the authors think these findings translate to human biology?

2) Although the greater response in exercise tolerance in the OKC-HETw rats was consistent with increased mitochondrial respiration in the OKC-HETw rats, these differences were not due to the expression levels of representative proteins in individual mitochondrial complexes nor mtDNA copy number. Therefore, these findings suggest that mtDNA sequence variation may be responsible. The authors indicate that there are 94 nucleotides mtDNA differences between the OKCHETB and OKC-HETW rats. This report is incomplete without an examination of whether any of these variations alter the sequence of the oxidative phosphorylation/electron transport complexes which might play a role in these differences. For example, as was done in the Dunham-Snary et al., 2018 reference.

END OF COMMENTS

Wake Forest University
School of Medicine

February 21, 2025

Ref: Mitochondrial Haplotype and Sex Modulate Adaptive Responses to Endurance Exercise Training

Dear editor,

We thank the reviewers and editors for their thorough and insightful evaluation of our manuscript, and we are thankful for the opportunity to submit our revised manuscript. Below you will find our detailed responses to the reviewers along with summaries of changes made to the manuscript on the basis of reviewer feedback.

Reviewers' Comments to Author:

Referee #1:

JP-RP-2024-288330 J Physiol: Mitochondrial Haplotype and Sex Modulate Adaptive Responses to Endurance Exercise Training

This manuscript highlights the relevance of mt genetic diversity and biological sex for understanding the variability of physiological responses to endurance training using OKC-HET$^{B/W}$ rats. Exercise tolerance increased to a greater extent in OKC-HET^{W} than OKC-HET^{B} rats. This is a fundamental contribution to evaluate the use of animal models for extrapolation to humans.

Comments (major and minor):
'Exercise adaptation' (Abstract) or 'adaptation to endurance exercise' (Introduction) is a widely and ambiguously used term and should be re-considered in an evolutionary and comparative physiological context. It is useful to restrict the term 'adaptation' to an evolutionary time scale, in contrast to 'exercise acclimation' studied at an immediate time scale; ref Hochachka PW, Somero GN (2002) Biochemical adaptation: mechanism and process in physiological evolution. Oxford Univ Press, New York: 466 pp. If 'acclimation' is defined as an immediate time scale adaption expressing phenotypic plasticity in response to changes of a single variable under controlled laboratory conditions, then 'adaption' may be considered as the general term, perhaps less technically compared to acclimation or even acclimatization. Following a concept of rigorously defined terms is particularly helpful in the present context when 'a large portion of exercise response variability is attributable to genetic heritability'.
- We replaced the term adaptation with acclimation where necessary throughout the manuscript. Additionally, several instances of “adaptive responses” were revised to “responses.”

p. 3: 'maximum oxygen consumption rate (OCR)' - as used here, is this equivalent to V_{O2max}, or what is the difference?
-Yes, maximal OCR is equivalent to VO2max. We modified the text as “Maximal OCR (i.e., VO2max)” to be clear for readers familiar with either term.

“Combining the anti-diabetic drug metformin with endurance exercise training blunted the improvements in the maximum oxygen consumption rate (OCR) or VO_{2max}, insulin sensitivity…”

p. 3: 'mitochondria are .. responsible for producing most of the cellular energy' and 'energy production' (Introduction) - The concept of 'energy production' contradicts the First Law of thermodynamics. I recommend the substitute 'transforming' for 'producing'.
-Thank you for pointing this out. We substituted the producing with transforming in page 3 shown below.

"Exercise training induces changes to the mitochondria (Oliveira & Hood, 2019), allowing for greater energy transformation during physical activity and improved endurance capacity."

p. 4: 'Maximal OCR of humans (Bassett & Howley, 2000) is significantly closer to that of rats (Bedford et al., 1979; Rodrigues et al., 2007) than of mice (Fernando et al., 1993; Schefer & Talan, 1996).' - It is necessary to give the units of the measure of OCR and refer to the underlying concept of scaling, to clarify the basis of comparing OCR in humans, rats, and mice. Is the OCR of humans closer to that of rats on the respiratory physiology or mitochondrial level, or both?
-The VO2max was measured on maximal oxygen consumption rate in humans and rodents. The VO2max in humans ranges from 30–60 ml/kg/min (Bassett), in rats from 60–80 ml/kg/min (Bedford; Rodrigues), and in mice from 140–180 ml/kg/min (Fernando & Schefer). We edited the text as below.

"Maximal OCR (i.e., VO_{2max}) in humans ranges from 30 to 60 ml/kg/min (Bassett & Howley, 2000), which is significantly closer to that of rats, ranging from 60 to 80 ml/kg/min (Bedford *et al.*, 1979; Rodrigues *et al.*, 2007) than of mice which have a VO_{2max} of 140-180 ml/kg/min (Fernando *et al.*, 1993; Schefer & Talan, 1996)."

p. 5, 9, 11, and several figures: 'weight' - must be replaced by 'mass'. The Bureau International des Poids et Mesures (2019) The International System of Units (SI). 9th edition:117-216 declares 'the necessity to put an end to the ambiguity which in current practice still exists on the meaning of the word *weight*, used sometimes for *mass*, sometimes for *mechanical force* .. The word "weight" denotes a quantity of the same nature as a "force": the weight of a body is the product of its mass and the acceleration due to gravity'.
-We changed weight to mass throughout the document.

p. 6: 'rats were sacrificed by carbon dioxide' - this may need a more detailed description.
-We provided more details about the euthanasia procedure as follows on page 6.
"…rats were sacrificed by carbon dioxide asphyxiation at a fill rate of 40-50% displacement of the chamber per minutes. After death was confirmed via cervical dislocation and toe pinching using forceps, laparotomy was performed for vital tissue dissections."

Typo p. 7: '10mM Ca-EGTA buffer - insert space: 10 mM
- We modified the text accordingly.

p. 7: 'Van Remmen et al., 2019' - The reference 'Van Remmen H, Pharaoh G & Ahn B. (2019). The Role of Mitochondrial Peroxide Release in the Mechanisms Underlaying Age-Related Sarcopenia.

Faseb Journal 33.' (p. 22) is incomplete and could not be found in PubMed.
Thank you for pointing this out. We replaced the reference.

p. 7: 'previously described method (Ahn et al., 2019 ..) - The protocol described in this reference is entirely different from the protocol used in the present manuscript. 'Rates of respiration were determined using sequential additions of substrates and inhibitors as follows: octanoylcarnitine (0.5 mM), malate (0.1 mM), ADP (2.5 mM), glutamate (10 mM), malate (2 mM), succinate (10 mM), rotenone (0.5 µM), Antimycin A (2.5 µM), and TMPD (0.5 mM) immediately followed by ascorbate (5 mM).' The rationale for this protocol is not explained, and references to the original publication(s) describing essential sections of this protocol are missing. Why is malate used sequentially at 0.1 mM and 2 mM? See https://doi.org/10.1152/ajpendo.00475.2015 and https://doi.org/10.1016/j.bbadis.2024.167544. Why is cytochrome c not added, which may be required for full CIV activity? How was oxygen consumption after addition of TMPD (0.5 mM) and ascorbate (5 mM) corrected for autoxidation of ascorbate and TMPD? If no corrections were applied, the 'CIV' data are artefacts and should be excluded from the results section.
- We modified our protocols from previous publications, but a reference published by Doerrier et al (Doerrier *et al.*, 2018) directly provides rationale for the substrate and inhibitor titration protocols. The first titration of malate (0.1 mM) is required for activation of FAO pathway. The second titration of malate (2 mM) is to activate NADH-linked respiration (i.e., complex 1).
- We acknowledge the concerns about autoxidation of TMPD. We excluded complex IV-induced respiration data from our results.
- About adding cytochrome c for activation of complex IV, we do not add it because diseases or damage to the mitochondria can lead to cytochrome release. Adding cytochrome c per se might mask the changes to the mitochondria between damaged and healthy mitochondria.

p. 7: 'All respiration measurements were normalized to Antimycin A to account for non-mitochondrial oxygen consumption.' - The term 'normalization' is confused with 'baseline correction'. 'Non-mitochondrial oxygen consumption' suggests that inhibition of the electron transfer system by Antimycin A inhibits all other oxygen-consuming processes in mitochondria. To correct for these ambiguities, the sentence should be written as: 'All respiratory rates were corrected for residual oxygen consumption measured after inhibition of the mitochondrial electron transfer system by Antimycin A.'
-We modified the text as follows.
"All respiratory rates were corrected for residual oxygen consumption measured after inhibition of the mitochondrial electron transport system by Antimycin A."

p. 7: While mitochondrial respiration was measured at 37C, measurement of force-frequency EDL muscle was performed at 32C. The rationale for this difference should be explained.
- We tested contractile properties of limb muscle at 32°C, given the temperature of human lower limb muscles (Chiesa *et al.*, 2016). For respirometry assays, the assays are historically performed at 37°C (Gnaiger, 2009) that allow comparisons with results from other groups (Kuznetsov *et al.*, 2008; Picard *et al.*, 2010). However, we acknowledge the discrepancy between contractile and mitochondrial experiments. Measuring the contractile and mitochondrial function at the same temperature (32°C) will be better study design for lower limb muscle. We will consider performing mitochondrial assays at closer temperatures of the tissue of interest in future studies. Thank you for your comments. We modified our text below.

We added our texts as follows:

"Measurements were performed on permeabilized fibers in MiR05 media at 37 ˚C, a temperature historically used in permeabilized myofiber experiments (Gnaiger, 2009) that allows comparisons with results from other studies (Kuznetsov *et al.*, 2008; Picard *et al.*, 2010)."

"Muscles were allowed 10 min of thermal equilibration at 32ºC, a temperature close to lower limb muscle of rats (Chiesa *et al.*, 2016).

p. 8: '**p* < .05'. See: Amrhein V, Greenland S, McShane B (2019) Scientists rise up against statistical significance. Nature 567:305-307.
Thank you for sharing the article. I have also been thinking about the discrepancy between dichotomy of statistical significance and continuous nature of numerical data. We used *for statistical significance but also showed p values throughout the manuscript.

p. 9: 'no significant difference was found in the running distance between the OKC-HET^B and OKC-HET^W rats within .. male (Fig. 1D) rats.' - Is the apparent (small) difference between the male haplotypes at weeks 1 to 5, and it's disappearance at weeks 7 to 8 of any interest?
- The running distance between OKC-HET^B and OKC-HET^W rats was similar with p values higher than 0.98 throughout.

p. 10: 'We sequentially added substrates that activate fatty acid oxidation, complex I, complex II, and complex IV.' - For the non-specialist, this terminology may be misleading. After addition of substrates (see p. 7) glutamate (10 mM) and malate (2 mM), the following respiratory Complexes are stimulated: CI, CIII, and CIV. After further addition of succinate and rotenone, the following respiratory Complexes are stimulated: CII, CIII, and CIV. 'Activation' in enzyme kinetics is to be distinguished from 'stimulation' by simply adding a substrate or, on the contrary, an inhibitor.
-Thank you for pointing this out. We replaced "activate" with "stimulate" on page 10. We added a reference in methods (Doerrier *et al.*, 2018) that provides rationale and complexes that are stimulated by our substrates and inhibitor titration protocol.

Figure 5 A and B: The coupling state LEAK requires further explanation in terms of pathway state. In the present SUIT protocol, it is the state (rate) FAO_L (or F_L). After addition of ADP (2.5 mM), the respiratory state is not 'State 3', but OXPHOS capacity at saturating ADP concentration (https://doi.org/10.26124/bec:2020-0001.v1). Compared to FAO (the pathway of fatty acid oxidation; strictly acylcarnitine oxidation when octanoylcarnitine plus malate 0.1 mM are used), C-1 is misleading (it is not CI activity) and is more correctly abbreviated as CI-linked or in terms of substrate&inhibitor-defined pathway capacity simply abbreviated as N (with NADH-linked substrates), and then NS (instead of C-I&II). C-I(Rot) is wrong: After inhibition of CI by rotenone, the pathway capacity is CII-linked (not C-I), simply indicated as S (succinate). CIV is in the present protocol CIV-OXPHOS capacity (if corrected for O2 concentration-dependent autoxidation of ascorbate and TMPD, and if not limited by cytochrome c). See https://doi.org/10.26124/bec:2020-0002 .
- We agree with the reviewer with the issues of the terminology. We modified our figure and texts accordingly. We appreciate the reviewer for pointing this out.

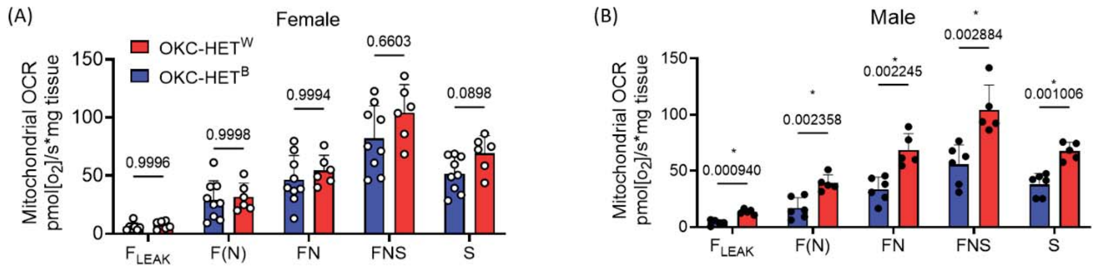

We modified our methods as below.

Rates of respiration were measured using sequential additions of substrates and inhibitors as follows: octanoylcarnitine (0.5 mM), malate (0.1 mM), ADP (2.5 mM), glutamate (10 mM), malate (2 mM), succinate (10 mM), rotenone (0.5 μM), Antimycin A (2.5 μM). Our experiments are designed to stimulate specific pathways that drive mitochondrial oxygen consumption, including fatty acid oxidation- (F), NADH- (N), FADH- (F) pathways as described previously (Doerrier *et al.*, 2018).

Typo p. 10: 'no significant differences were observed between the two mt haplotypes in male rats (Fig. 8B). - This should be Fig. 9B.
We modified our text accordingly.

Figure 9F: The data look very different from $^{**}P < .01$. It is more convincing to give the actual *P*-value (see reference suggested for p. 8).
-We provided p values for the comparisons whether the values reached statistical significance or not throughout the manuscript. Please see figures.

p. 11: 'Consistent with previous studies, we observed that female rodents run significantly more than males (Jones et al., 1990; Rosenfeld, 2017)' - The references could be extended to include more results on mice: https://doi.org/10.1152/ajpregu.00694.2005, https://doi.org/10.1590/1414-431X20187830
-Thanks you for your suggestion. The reference is added in the text.

Referee #2:

This manuscript addresses a very important characteristic when using rodent models in an attempt to understand the role of genetics in exercise response variability, that many investigators may not be aware of. That is, most investigations using rodent models use inbred strains, which have no mitochondrial diversity, because each inbred strain of laboratory mice originates from a single female mouse, and hence each mouse studied has the same mitochondrial genetics. The investigators utilized a novel OKC-HET rat model, which has 94 nucleotides mtDNA differences between the OKCHETB and OKC-HETW rats, to investigate the impact of mt haplotype on exercise training (voluntary wheel running).

One of the most striking findings was that females engaged in approximately 5-8 times as much voluntary wheel running than males. However, wheel running distance was not affected by mtDNA differences.

Exercise tolerance increased in both male and female rats in response to endurance exercise training. There was a significant effect of mtDNA haplogroup, with the increased endurance being greater in the OKC-HETw female rats, and only apparent in males with the OKC-HETw mtDNA haplogroup. Consistent with the greater response in the OKC-HETw rats, mitochondrial respiration was higher in the OKC-HETw rats. However, neither the expression of representative proteins in individual mitochondrial complexes nor mtDNA copy number played a role in these mtDNA haplogroup differences since these were not different by mtDNA haplogroup.

One parameter usually associated with negative health outcomes, mtDNA deletion frequency was higher in the OKC-HETw rats. However, 4-HNE, a marker of lipid peroxidation was lower in the female OKC-HETw rats.

Specific Comments

1) Regarding the strikingly higher wheel running in the female rats:
a. Do the authors think that the outcomes would be different if they instituted a similar exercise training level in the males and females? And would that have been a better approach?
- Matching exercise training volume by using treadmill running is one possible approach. However, this method lacks the voluntary nature of wheel running, which is a critical factor in our study design. Forced exercise had an adverse impact on the lifespan of rats (Narath *et al.*, 2001). Given the similar VWR activities at younger ages, we could help minimize the differences in VWR activities while preserving the voluntary aspect of exercise.

Our response is addressed in the second paragraph of the discussion as follows: "Endurance exercise in rodents is commonly conducted using either running wheels or treadmills. In this study, we selected running wheels to investigate training effects because they allow for voluntary activity, in contrast to forced exercise imposed by treadmill running that have an impact on lifespan (Narath *et al.*, 2001). Our approach more accurately reflects the voluntary nature of human exercise."

b. How do the authors reconcile the body weight loss after exercise, with no weight loss in females, but significant weight loss in males?
- Given the p value of body mass changes after VWR activities in female rats ($p < 0.14$; Fig 2D), we think there is a trend toward decreased body mass, which did not reach statistical significance due to limited sample size. The effect of VWR on the body mass appeared more pronounced in males, partially due to greater fat mass, as male rats are ~85% heavier than females (Fig 2A). Overall, we think that the endurance exercise has effects on body mass for both sexes, but the effects are more substantial in males. We modified our text as follows (page 9).

"We found that the female rats showed a trend toward decreased body mass after the eight-week endurance training ($p < 0.14$; Fig. 2D). Male rats, despite running much less than the females, lost 2.5-5% of their baseline body mass after training (Fig. 2E), showing greater efficacy for training responses for male rats than females."

c. In light of the extreme sex difference in exercise training and lack of concordant weight loss, how do the authors think these findings translate to human biology?

Men seem to more sensitive to exercise training than women in line with our data in OKC-HET rats. In a randomized controlled clinical trial comparing body mass changes of overweight and obese men and women, only men in the exercise group lost body mass (~5kg), while men in the control did not lose or gain body mass. Women in the exercise group had a small increase in body mass (0.6 kg), while the control group had a greater increase in body mass gain (~3 kg). Thus, our results from OKC-HET rats are in line with the findings from humans. The considerable difference in voluntary activity is a limitation of our study, which we addressed in results (page 9) as follows.

"We found that the female rats showed a trend toward decreased body mass after the eight-week endurance training ($p < 0.14$; Fig. 2D). Male rats, despite running much less than the females, lost 2.5-5% of their baseline body mass after training (Fig. 2E), showing greater efficacy for training responses for male rats than females. Our results are in line with results from humans (Donnelly *et al.*, 2003). In a randomized controlled clinical trial comparing 16 months exercise effects on body mass changes of overweight and obese men and women, only men in the exercise group lost body mass (~5kg), while men in the control did not lose or gain body mass. Women in the exercise group had a small increase in body mass (0.6 kg), while the control group had a greater increase in body mass gain (~3 kg). Thus, higher sensitivity in body mass loss in male rats is in line with the findings from humans."

2) Although the greater response in exercise tolerance in the OKC-HETw rats was consistent with increased mitochondrial respiration in the OKC-HETw rats, these differences were not due to the expression levels of representative proteins in individual mitochondrial complexes nor mtDNA copy number. Therefore, these findings suggest that mtDNA sequence variation may be responsible. The authors indicate that there are 94 nucleotides mtDNA differences between the OKCHETB and OKC-HETW rats. This report is incomplete without an examination of whether any of these variations alter the sequence of the oxidative phosphorylation/electron transport complexes which might play a role in these differences. For example, as was done in the Dunham-Snary et al., 2018 reference.
-We examined the nucleotide variants in our first paper on the OKC-HET rats (Sathiaseelan *et al.*, 2023). 16 of the nucleotide differences are nonsynonymous substitutions in the genes for ND2, COI, ATPase8, ATPase6, ND4, ND6, and Cytb.

We modified our text to add the information in the conclusion (page 14) as follows:
"In summary, our data shows for the first time that the mt haplotype significantly impacts training responses to endurance exercise in a sex-specific manner. Using OKC-HET$^{B/W}$ rats, we demonstrated that exercise-induced changes are mitochondrial haplotype-specific, with some phenotypes manifesting in one sex but not the other. Our previous work identified 94 nucleotide differences between the two mitochondrial genomes, including sixteen nonsynonymous substitutions in the genes for ND2, COI, ATPase8, ATPase6, ND4, ND6, and Cytb (Sathiaseelan *et al.*, 2023). OKC-HET rats may serve as a model for testing the functional significance of specific mtDNA variants in endurance training responses."

Reviewing Editor:
Thank you for submitting your work to The Journal of Physiology. Whereas both reviewers were enthusiastic about the novelty of the work, both had comments that will need to be addressed in a revision. Reviewer 1 was primarily concerned with accuracy in terminology and makes some good points concerning the mitochondrial substrate-inhibitor protocol that was employed. Reviewer 2's main request was additional information about what is encoded by the 94 nucleotides that differ between the two rat strains. This should be relatively easy to determine and should be provided by the authors in their revision. An additional question concerns the rationale for studying 18-19 mo old animals. Are the survival characteristics of the rats with the different mitochondrial haplotypes known and could differences in the % survival between them contribute to the differences between strains that were observed. For example, if one strain were biologically older (at a lower %survival) despite being the same absolute age (in months), this could presumably affect the amount of voluntary activity and the nature of the muscle alterations. Further to this point, since male rats tend to show a considerably greater increase in body fat with increasing age than females (see Turturro et al. J Gerontol Biol Sci Med Sci 54[11]: B492-501, 1999), this is a likely contributor to the differences in voluntary activity between male and female rats and may not have been as extreme at younger ages. This point should be addressed by the authors in their revision.

-Regarding the information about the 94 nucleotides, we reported the nucleotide variants in our first paper on the OKC-HET rats (Sathiaseelan *et al*., 2023). 16 of the nucleotide differences are nonsynonymous substitutions in the genes for ND2, COI, ATPase8, ATPase6, ND4, ND6, and Cytb. We modified our text in conclusion as below.

"Our previous work identified 94 nucleotide differences between the two mitochondrial genomes, including sixteen nonsynonymous substitutions in the genes for ND2, COI, ATPase8, ATPase6, ND4, ND6, and Cytb (Sathiaseelan *et al.*, 2023). OKC-HET rats may serve as a model for testing the functional significance of specific mtDNA variants in endurance training responses."

-Regarding strain differences in the life span, our experiments led by Dr. Arlan Richardson are currently underway. If one strain lives longer than the other, we agree that this can help explain the exercise acclimation differences.

-We also agree with the comment about the fat mass being contributor to the sex differences in the voluntary activity of OKC-HET rats. We also think that greater fat mass in the male rats plays a role in the sex differences. We modified our text as below.

"A limitation of our study is the significant sex differences in voluntary activities. Male rats show the grater increases in body mass and fat with increasing age than females (Turturro *et al.*, 1999; Narath *et al.*, 2001), which likely contributes to the differences in the voluntary activities between sexes. While our study used older animals with focuses on the effects of endurance training on sarcopenia, younger animals generally exhibit higher activities than older ones (Narath *et al.*, 2001) and expected to exhibit more similar voluntary activities across sexes than older animals.

Senior Editor:

Comments for Authors to ensure the paper complies with the Statistics Policy:
Exact p values not used, data shown as mean +/- SEM, not SD.

Comments to the Author:
Thank you for the Manuscript submission to The Journal of Physiology. It has been considered by a reviewing editor and two expert reviewers. All were positive about the work but have raised a number of important points that need to be considered by the authors. In addition, in the "study design" section of the Methods please state the total number of animals used. This is important because of the different animal numbers used in study groups depicted in the Figures. Please also ensure the manuscript complies to the Journal statistics policy, i.e. please use exact p values and present data as mean +/- SD, not SEM throughout. We look forward to receiving the revised manuscript. Thank you for considering The Journal of Physiology.

We included the number of rats we used from each group in the methods section as follows. “We used a total of 71 rats (35 males and 36 females). Among the males, 20 were of the OKC-HETB strain, and 15 were of the OKC-HETW strain. Among the females, 22 were of the OKC-HETB strain, and 14 were of the OKC-HETW strain.”

Also, we modified our text throughout using exact p values and SD.

References

Bassett DR & Howley ET. (2000). Limiting factors for maximum oxygen uptake and determinants of endurance performance. *Medicine and Science in Sports and Exercise* **32,** 70-84.

Bedford TG, Tipton CM, Wilson NC, Oppliger RA & Gisolfi CV. (1979). Maximum Oxygen-Consumption of Rats and Its Changes with Various Experimental Procedures. *J Appl Physiol* **47,** 1278-1283.

Chiesa ST, Trangmar SJ & Gonzalez-Alonso J. (2016). Temperature and blood flow distribution in the human leg during passive heat stress. *J Appl Physiol (1985)* **120,** 1047-1058.

Doerrier C, Garcia-Souza LF, Krumschnabel G, Wohlfarter Y, Meszaros AT & Gnaiger E. (2018). High-Resolution FluoRespirometry and OXPHOS Protocols for Human Cells, Permeabilized Fibers from Small Biopsies of Muscle, and Isolated Mitochondria. *Methods Mol Biol* **1782,** 31-70.

Donnelly JE, Hill JO, Jacobsen DJ, Potteiger J, Sullivan DK, Johnson SL, Heelan K, Hise M, Fennessey PV, Sonko B, Sharp T, Jakicic JM, Blair SN, Tran ZV, Mayo M, Gibson C & Washburn RA. (2003). Effects of a 16-month randomized controlled exercise trial on body weight and composition in young, overweight men and women: the Midwest Exercise Trial. *Arch Intern Med* **163,** 1343-1350.

Fernando P, Bonen A & Hoffman-Goetz L. (1993). Predicting submaximal oxygen consumption during treadmill running in mice. *Can J Physiol Pharmacol* **71,** 854-857.

Gnaiger E. (2009). Capacity of oxidative phosphorylation in human skeletal muscle: new perspectives of mitochondrial physiology. *Int J Biochem Cell Biol* **41,** 1837-1845.

Kuznetsov AV, Veksler V, Gellerich FN, Saks V, Margreiter R & Kunz WS. (2008). Analysis of mitochondrial function in situ in permeabilized muscle fibers, tissues and cells. *Nat Protoc* **3,** 965-976.

Narath E, Skalicky M & Viidik A. (2001). Voluntary and forced exercise influence the survival and body composition of ageing male rats differently. *Exp Gerontol* **36,** 1699-1711.

Oliveira AN & Hood DA. (2019). Exercise is mitochondrial medicine for muscle. *Sports Med Hlth Sci* **1,** 11-18.

Picard M, Ritchie D, Wright KJ, Romestaing C, Thomas MM, Rowan SL, Taivassalo T & Hepple RT. (2010). Mitochondrial functional impairment with aging is exaggerated in isolated mitochondria compared to permeabilized myofibers. *Aging Cell* **9,** 1032-1046.

Rodrigues B, Figueroa DM, Mostarda CT, Heeren MV, Irigoyen MC & De Angelis K. (2007). Maximal exercise test is a useful method for physical capacity and oxygen consumption determination in streptozotocin-diabetic rats. *Cardiovasc Diabetol* **6**.

Sathiaseelan R, Ahn B, Stout MB, Logan S, Wanagat J, Nguyen HVM, Hord NG, Vandiver AR, Selvarani R, Ranjit R, Yarbrough H, Masingale A, Miller BF, Wolf RF, Austad SN & Richardson A. (2023). A Genetically Heterogeneous Rat Model with Divergent Mitochondrial Genomes. *J Gerontol A Biol Sci Med Sci* **78,** 771-779.

Schefer V & Talan MI. (1996). Oxygen consumption in adult and AGED C57BL/6J mice during acute treadmill exercise of different intensity. *Exp Gerontol* **31,** 387-392.

Turturro A, Witt WW, Lewis S, Hass BS, Lipman RD & Hart RW. (1999). Growth curves and survival characteristics of the animals used in the Biomarkers of Aging Program. *J Gerontol A Biol Sci Med Sci* **54,** B492-501.

Dear Dr Ahn,

Re: JP-RP-2025-288330R1 "Mitochondrial Haplotype and Sex Modulate Responses to Endurance Exercise Training" by Bumsoo Ahn, Tianhao Wei, Ryan Pettit-Mee, Eunyoung Kim, Robert Vincent Musci, Jonathan Wanagat, Hoang Van Nguyen, Arlan Richardson, and Hyunyoung Kim

Thank you for submitting your manuscript to The Journal of Physiology. It has been assessed by a Reviewing Editor and by 1 expert referee and we are pleased to tell you that it is acceptable for publication following satisfactory revision.

The referee reports are copied at the end of this email.

Please address all the points raised and incorporate all requested revisions or explain in your Response to Referees why a change has not been made. We hope you will find the comments helpful and that you will be able to return your revised manuscript within 4 weeks. If you require longer than this, please contact journal staff: jp@physoc.org.

Your revised manuscript should be submitted online using the link in your Author Tasks Link Not Available. This link is accessible via your account as Corresponding Author; it is not available to your co-authors. If this presents a problem, please contact journal staff (jp@physoc.org). Image files from the previous version are retained on the system. Please ensure you replace or remove any files that are being revised.

If you do not wish to submit a revised version of your manuscript, you must inform our journal staff (jp@physoc.org) or reply to this email to request withdrawal. Please note that a manuscript must be formally withdrawn from the peer review process at one journal before it may be submitted to another journal.

TRANSPARENT PEER REVIEW POLICY: To improve the transparency of its peer review process The Journal of Physiology publishes online, as supporting information, the peer review history of all articles accepted for publication. Readers will have access to decision letters, including Editors' comments and referee reports, for each version of the manuscript, as well as any author responses to peer review comments. Referees can decide whether or not they wish to be named on the peer review history document.

ABSTRACT FIGURES: Authors are expected to use The Journal's premium BioRender account to create/redraw their Abstract Figures. Information on how to access this account is here: https://physoc.onlinelibrary.wiley.com/journal/14697793/biorender-access.

This will enable Authors to create and download high-resolution figures. If authors have used the free BioRender service, they can use the instructions provided in the link above to download a high-resolution version suitable for publication.
The link provided should only be used for the purposes of this submission. Authors will be charged for figures created on this account if they are not related to this manuscript submission.

LANGUAGE EDITING AND SUPPORT FOR PUBLICATION: If you would like help with English language editing, or other article preparation support, Wiley Editing Services offers expert help, including English Language Editing, as well as translation, manuscript formatting, and figure formatting at www.wileyauthors.com/eeo/preparation. You can also find resources for Preparing Your Article for general guidance about writing and preparing your manuscript at www.wileyauthors.com/eeo/prepresources.

RIGOUR AND REPRODUCIBILITY: We recommend authors consult The Journal's Rigour and Reproducibility requirements to ensure that their manuscript is suitable for publication: https://physoc.onlinelibrary.wiley.com/pb-assets/hub-assets/physoc/documents/TJP-Rigour-and-Reproducibility-Requirements.pdf

REVISION CHECKLIST:
Check that your Methods section conforms to journal policy: https://jp.msubmit.net/cgi-bin/main.plex?form_type=display_requirements#methods

Check that data presented conforms to the statistics policy: https://jp.msubmit.net/cgi-bin/main.plex?form_type=display_requirements#statistics

Upload a full Response to Referees file. To create your 'Response to Referees' copy all the reports, including any comments from the Senior and Reviewing Editors, into a Microsoft Word, or similar, file and respond to each point, using font or background colour to distinguish comments and responses and upload as the required file type.

Please upload two versions of your manuscript text: one with all relevant changes highlighted and one clean version with no changes tracked. The manuscript file should include all tables and figure legends, but each figure/graph should be uploaded as separate, high-resolution files. The journal is now integrated with Wiley's Image Checking service. For further details, see: https://www.wiley.com/en-us/network/publishing/research-publishing/trending-stories/upholding-image-integrity-wileys-image-screening-service

You may also upload:
- 'Potential Cover Art' for consideration as the issue's cover image
- Appropriate Supporting Information (Video, audio or data set: see https://jp.msubmit.net/cgi-bin/main.plex?form_type=display_requirements#supp).

We look forward to receiving your revised submission.

If you have any queries, please reply to this email and we will be pleased to advise.

Yours sincerely,

Paul Greenhaff
Senior Editor
The Journal of Physiology

REQUIRED ITEMS

(1) Your manuscript must include a complete Additional Information section, including competing interests; funding; author contributions and acknowledgements.
[**COMPETING INTERESTES STATEMENT IS MISSING**]

(2) Please upload separate high-quality figure files via the submission form. [**ONE FILE FOR EACH FIGURE PLEASE**]

(3) Papers must comply with the Statistics Policy: https://jp.msubmit.net/cgi-bin/main.plex?form_type=display_requirements#statistics.

In summary:

- If n â‰¤ 30, all data points must be plotted in the figure in a way that reveals their range and distribution. A bar graph with data points overlaid, a box and whisker plot or a violin plot (preferably with data points included) are acceptable formats.

- If n > 30, then the entire raw dataset must be made available either as supporting information, or hosted on a not-for-profit repository, e.g. FigShare, with access details provided in the manuscript.

- 'n' clearly defined (e.g. x cells from y slices in z animals) in the Methods. Authors should be mindful of pseudoreplication.

- All relevant 'n' values must be clearly stated in the main text, figures and tables.

- The most appropriate summary statistic (e.g. mean or median and standard deviation) must be used. Standard Error of the Mean (SEM) alone is not permitted.

- Exact p values must be stated. Authors must not use 'greater than' or 'less than'. Exact p values must be stated to three significant figures even when 'no statistical significance' is claimed.

(4) Please include an Abstract Figure file, as well as the Figure Legend text within the main article file.
[**WE HAVE THE FIGURE, BUT THE LEGEND IS MISSING**]
The Abstract Figure is a piece of artwork designed to give readers an immediate understanding of the research and should summarise the main conclusions. If possible, the image should be easily 'readable' from left to right or top to bottom. It should show the physiological relevance of the manuscript so readers can assess the importance and content of its findings. Abstract Figures should not merely recapitulate other figures in the manuscript. Please try to keep the diagram as simple as possible and without superfluous information that may distract from the main conclusion(s). Abstract Figures must be provided by authors no later than the revised manuscript stage and should be uploaded as a separate file during online submission labelled as File Type 'Abstract Figure'. Please also ensure that you include the figure legend in the main article file. All Abstract Figures should be created using BioRender. Authors should use The Journal's premium BioRender account to export high-resolution images. Details on how to use and access the premium account are included as part of this email.

EDITOR COMMENTS

Reviewing Editor:

Thank you for sending your revised work. Whilst the manuscript is substantially improved, there remain two minor issues for you to address as below:

1. Please check P 1 in the text that has been revised. Here you state "maximal oxygen consumption rate (OCR) or VO2max". OCR is a term that is typically used in studies examining mitochondrial respiration (e.g., Seahorse platform), whereas VO2max is typically whole body exercise. As such, I suggest you simply say "maximal rate of oxygen consumption (VO2max)". Further to this point, later in this same statement you use the term "aerobic capacity" and this is often used to represent VO2max, which then makes the former mention of VO2max redundant. Please clarify this statement.

2. The authors should provide some explanation for why the female mice did not exhibit a significant body mass reduction with the voluntary exercise, despite a considerably greater volume of activity than the males who did lose body mass with the voluntary exercise. For example, did the females increase their caloric intake to compensate for the increase in activity whereas the males did not?

Please also see 'Required Items' above.

Senior Editor:

Comments for Authors to ensure the paper complies with the Statistics Policy (Required):
The statistical analysis section of the Methods states values represent mean +/- SM? Presumably this is a typographical error and should be SD as the figure legends state SD.

Please use exact P values throughout, not P<

The legend to Figs 2, 4, 5, 6, 7, 8 and 9 all state *P<0.05, but it looks like both * and exact P values are presented in each figure. This is confusing. Please use only exact p values and amend the legend of each figure to convey this.

Comments to the Author:
Thank you for the revised manuscript submission which has been considered by the same reviewers that considered the original submission. There is agreement that the manuscript has been substantially improved but several points remain that need to be addressed by the authors. The formatting of the manuscript also needs some attention so as to align with Journal of Physiology guidelines. We look forward to receiving the revised version.

REFEREE COMMENTS

Referee #2:

The authors have made many changes that addressed both reviewers' concerns.

However, the original concern regarding the lack of weight loss in females, in spite of the fact that females engaged in 5-8 times as much voluntary wheel running while males lost significant body weight (2.5-5%). The explanation that there was a trend for weight loss in females, and that show greater efficacy for training responses for male rats than females is inadequate. Some discussion of what the differences in efficacy for training responses might be is in order, eg. Decreased intake in females in response to exercise, or other explanations.

END OF COMMENTS

Wake Forest University
School of Medicine

April 13, 2025

Ref: Mitochondrial Haplotype and Sex Modulate Adaptive Responses to Endurance Exercise Training

Dear editor,

We thank the editors and reviewer 2 for their suggestions of our manuscript. Below you will find our responses along with the changes made to the manuscript on the basis of reviewer feedback.

Reviewing Editor
1. Please check P 1 in the text that has been revised. Here you state "maximal oxygen consumption rate (OCR) or VO2max". OCR is a term that is typically used in studies examining mitochondrial respiration (e.g., Seahorse platform), whereas VO2max is typically whole body exercise. As such, I suggest you simply say "maximal rate of oxygen consumption (VO2max)". Further to this point, **later in this same statement you use the term "aerobic capacity" and this is often used to represent VO2max, which then makes the former mention of VO2max redundant. Please clarify this statement.**

➔ Thank you for your comment. We modified our text as follows:
"Combining the anti-diabetic drug metformin with endurance exercise training blunted the improvements in the maximum oxygen consumption (VO2max), insulin sensitivity, and aerobic capacity, elicited by endurance exercise."

2. The authors should provide some explanation for why the female mice did not exhibit a significant body mass reduction with the voluntary exercise, despite a considerably greater volume of activity than the males who did lose body mass with the voluntary exercise. For example, did the females increase their caloric intake to compensate for the increase in activity whereas the males did not?

- ➔ We anticipate the lack of body mass reductions following exercise in female rats may have resulted from hormonal changes at 20-21 months of age. Another contributing factor may be sex-specific differences in the effects of endurance exercise on food intake, as only male rats exhibited reduced food consumption, whereas female rats increased their intake. We modified our text below.
- ➔ "We found that the female rats showed a trend toward decreased body mass after the eight-week endurance training (P=0.1396; Fig. 2D; n=17 rats). Male rats, despite running much less than the females, lost 2.5-5% of their baseline body mass after training (Fig. 2E, n=20 rats), showing greater efficacy for training responses for male rats than females. The absence of body mass reduction following exercise training in female rats may be related to age-associated hormonal changes. At 20-21 months of age, alterations in ovarian function and the estrous cycle (i.e., changes in circulating estradiol and

progesterone) may attenuate the physiological responses to endurance training. Another contributing factor could be sex-specific differences in the effects of endurance exercise on food intake, as only male rats exhibited reduced food consumption, whereas female rats increased their intake (Foright *et al.*, 2020)."

Please also see 'Required Items'.
➔ We edited the texts and figures accordingly.

Referee #2:

The authors have made many changes that addressed both reviewers' concerns.

However, the original concern regarding the lack of weight loss in females, in spite of the fact that females engaged in 5-8 times as much voluntary wheel running while males lost significant body weight (2.5-5%). **The explanation that there was a trend for weight loss in females, and that show greater efficacy for training responses for male rats than females is inadequate. Some discussion of what the differences in efficacy for training responses might be is in order, eg. Decreased intake in females in response to exercise, or other explanations.**

➔ As discussed above, we anticipate that the hormonal changes in female rats and sex-specific differences in the effects of endurance exercise on food intake are potential contributing factors to the differential effects of endurance training on male and female rats. We modified our text as below.

"We found that the female rats showed a trend toward decreased body mass after the eight-week endurance training (P=0.1396; Fig. 2D; n=17 rats). Male rats, despite running much less than the females, lost 2.5-5% of their baseline body mass after training (Fig. 2E, n=20 rats), showing greater efficacy for training responses for male rats than females. The absence of body mass reduction following exercise training in female rats may be related to age-associated hormonal changes. At 20-21 months of age, alterations in ovarian function and the estrous cycle (i.e., changes in circulating estradiol and progesterone) may attenuate the physiological responses to endurance training. Another contributing factor could be sex-specific differences in the effects of endurance exercise on food intake, as only male rats exhibited reduced food consumption, whereas female rats increased their intake (Foright *et al.*, 2020)."

Reference

Foright RM, Johnson GC, Kahn D, Charleston CA, Presby DM, Bouchet CA, Wellberg EA, Sherk VD, Jackman MR, Greenwood BN & MacLean PS. (2020). Compensatory eating behaviors in male and female rats in response to exercise training. *Am J Physiol Regul Integr Comp Physiol* **319,** R171-R183.

2nd Revision - Editorial Decision 30-Apr-2025

Dear Dr Ahn,

Re: JP-RP-2025-288330R2 "Mitochondrial Haplotype and Sex Modulate Responses to Endurance Exercise Training" by Bumsoo Ahn, Tianhao Wei, Ryan Pettit-Mee, Eunyoung Kim, Robert Vincent Musci, Jonathan Wanagat, Hoang Van Nguyen, Arlan Richardson, and Hyunyoung Kim

We are pleased to tell you that your paper has been accepted for publication in The Journal of Physiology.

The last Word (or similar) version of the manuscript provided will be used by the Production Editor to prepare your proof. When this is ready you will receive an email containing a link to Wiley's Online Proofing System. The proof should be thoroughly checked and corrected as promptly as possible.

Authors should note that it is too late at this point to offer corrections prior to proofreading. Major corrections at proof stage, such as changes to figures, will be referred to the Editors for approval before they can be incorporated. Only minor changes, such as to style and consistency, should be made at proof stage. Changes that need to be made after proof stage will usually require a formal correction notice.

All queries at proof stage should be sent to: TJP@wiley.com.

Following copyediting and typesetting the accepted version will be published online.

Yours sincerely,

Paul Greenhaff
Senior Editor
The Journal of Physiology

IMPORTANT POINTS TO NOTE FOLLOWING YOUR PAPER'S ACCEPTANCE:

If you would like to receive our 'Research Roundup', a monthly newsletter highlighting the cutting-edge research published in The Physiological Society's family of journals (The Journal of Physiology, Experimental Physiology, Physiological Reports, The Journal of Nutritional Physiology and The Journal of Precision Medicine: Health and Disease), please click this link, fill in your name and email address and select 'Research Roundup':
https://www.physoc.org/journals-and-media/membernews

• TRANSPARENT PEER REVIEW POLICY: To improve the transparency of its peer review process, The Journal of Physiology publishes online as supporting information the peer review history of all articles accepted for publication. Readers will have access to decision letters, including Editors' comments and referee reports, for each version of the manuscript as well as any author responses to peer review comments. Referees can decide whether or not they wish to be named on the peer review history document.

• You can help your research get the attention it deserves! Check out Wiley's free Promotion Guide for best-practice recommendations for promoting your work at: www.wileyauthors.com/eeo/guide. You can learn more about Wiley Editing Services which offers professional video, design, and writing services to create shareable video abstracts, infographics, conference posters, lay summaries, and research news stories for your research at: www.wileyauthors.com/eeo/promotion.

• IMPORTANT NOTICE ABOUT OPEN ACCESS: To assist authors whose funding agencies mandate public access to published research findings sooner than 12 months after publication, The Journal of Physiology allows authors to pay an Open Access (OA) fee to have their papers made freely available immediately on publication.

The Corresponding Author will receive an email from Wiley with details on how to register or log-in to Wiley Authors Services where you will be able to place an order.

• You can check if your funder or institution has a Wiley Open Access Account here: https://authorservices.wiley.com/author-resources/Journal-Authors/licensing-and-open-access/open-access/author-compliance-tool.html.

EDITOR COMMENTS

Reviewing Editor:

Thank you for submitting your revised work to The Journal of Physiology. All prior concerns have now been addressed.

Senior Editor:

Thank you for revising the manuscript. The Reviewing Editor has indicated the authors have addressed the concerns that were previously identified and believes the manuscript is suitable for publication. Thank you for choosing The Journal of Physiology to publish your research.
